# Anastomosis Groups and Mycovirome of *Rhizoctonia* Isolates Causing Sugar Beet Root and Crown Rot and Their Sensitivity to Flutolanil, Thifluzamide, and Pencycuron

**DOI:** 10.3390/jof9050545

**Published:** 2023-05-09

**Authors:** Can Zhao, Siwei Li, Zhihao Ma, Wenjun Wang, Lihong Gao, Chenggui Han, Anpei Yang, Xuehong Wu

**Affiliations:** 1 College of Plant Protection, China Agricultural University, Beijing 100193, China; 2 College of Horticulture, China Agricultural University, Beijing 100193, China; 3 Institute of Plant Protection, Xinjiang Academy of Agricultural Science, Urumqi 830091, China

**Keywords:** sugar beet, root and crown rot, *Rhizoctonia*, anastomosis group, mycovirome, sensitivity, flutolanil, thifluzamide, pencycuron

## Abstract

Anastomosis groups (AGs) or subgroups of 244 *Rhizoctonia* isolates recovered from sugar beet roots with symptoms of root and crown rot were characterized to be AG-A, AG-K, AG-2-2IIIB, AG-2-2IV, AG-3 PT, AG-4HGI, AG-4HGII, and AG-4HGIII, with AG-4HGI (108 isolates, 44.26%) and AG-2-2IIIB (107 isolates, 43.85%) being predominate. Four unclassified mycoviruses and one hundred and one putative mycoviruses belonging to six families, namely *Mitoviridae* (60.00%), *Narnaviridae* (18.10%), *Partitiviridae* (7.62%), *Benyviridae* (4.76%), *Hypoviridae* (3.81%), and *Botourmiaviridae* (1.90%), were found to be present in these 244 *Rhizoctonia* isolates, most of which (88.57%) contained positive single-stranded RNA genome. The 244 *Rhizoctonia* isolates were all sensitive to flutolanil and thifluzamide, with average median effective concentration (EC_50_) value of 0.3199 ± 0.0149 μg·mL^−1^ and 0.1081 ± 0.0044 μg·mL^−1^, respectively. Among the 244 isolates, except for 20 *Rhizoctonia* isolates (seven isolates of AG-A and AG-K, one isolate of AG-4HGI, and 12 isolates of AG-4HGII), 117 isolates of AG-2-2IIIB, AG-2-2IV, AG-3 PT, and AG-4HGIII, 107 isolates of AG-4HGI, and six isolates of AG-4HGII were sensitive to pencycuron, with average EC_50_ value of 0.0339 ± 0.0012 μg·mL^−1^. Correlation index (*ρ*) of cross-resistance level between flutolanil and thifluzamide, flutolanil and pencycuron, and thifluzamide and pencycuron was 0.398, 0.315, and 0.125, respectively. This is the first detailed study on AG identification, mycovirome analysis, and sensitivity to flutolanil, thifluzamide, and pencycuron of *Rhizoctonia* isolates associated with sugar beet root and crown rot.

## 1. Introduction

Sugar beet (*Beta vulgaris* L.) is the second largest sugar crop worldwide, which is mainly cultivated in Xinjiang Uygur and Inner Mongolia autonomous regions, and Hebei, Heilongjiang, Gansu, Jilin, and Shanxi provinces of China [1]. Seedling damping-off, root and crown rot, foliar blight, as well as dry rot canker are common diseases that occur in the growing season or at the storage period of sugar beet, and all these diseases can be caused by the phytopathogen *Rhizoctonia* [2]. Of these diseases, sugar beet root and crown rot is the most serious and important disease, which causes decline in yield and saccharinity directly, and has an impact on storage of sugar beet [3,4,5].

It was widely known that anastomosis group (AG)-2-2IIIB and AG-2-2IV of *R. solani* were the predominant AGs causing sugar beet root and crown rot [5,6,7], while AG-4HGI, AG-4HGII, and AG-4HGIII were mainly attributed to sugar beet seedling damping-off [8,9,10]. Besides AG-2-2IIIB and AG-2-2IV, binucleate *Rhizoctonia* (BNR) (including AG-A, AG-D, AG-E, and AG-K) and multinucleate *Rhizoctonia* (MNR) (including AG-4HGI and AG-4HGII) were also occasionally isolated from sugar beet roots with symptoms of root and crown rot in Iran and the United States of America (USA) [7,11]. However, only two reports related to sugar beet root and crown rot had been documented in China; one was reported in our previous study that AG-2-2IIIB was the causal agent of sugar beet root and crown rot in Datong city of Shanxi province [12]; the other was recorded by Zhang et al. [13] that AG-4HGI was associated with sugar beet root and crown rot in Hohohot city of Inner Mongolia autonomous region.

Because of the broad host range of *Rhizoctonia* and the limitation of the high resistant commercial cultivars with high yield, management of sugar beet root and crown rot caused by *Rhizoctonia* mainly relied on the timely application of fungicides. It was reported that the quinone outside inhibitor (QoI), azoxystrobin, was the most widely used fungicide for controlling *R. solani* on sugar beet since its registration in USA in 1997 [14]. However, the resistance for *Rhizoctonia* isolates to azoxystrobin had been reported due to the long-time use of this fungicide [15,16]. In addition, the succinate dehydrogenase inhibitor (SDHI), penthiopyrad, was recorded to be able to effectively control *R. solani* on sugar beet in USA [17]. Flutolanil, polyoxin-D, and azoxystrobin were also documented to provide a high level of disease suppression to suppress sugar beet disease developed by AG-2-2IIIB and AG-2-2IV in the Red River Valley of USA [18]. There were only two fungicides, namely fenaminosulf and thiram, that had been registered in China for controlling sugar beet root and crown rot (China Pesticide Information Network, www.chinapesticide.gov.cn, accessed on 8 January 2021). Repeated use of fungicides with a single-site mode-of-action favors the development of resistance in fungal populations. Therefore, it is of importance to screen new fungicides for controlling sugar beet root and crown rot caused by *Rhizoctonia*.

Both flutolanil and thifluzamide are SDHI fungicides, which are effectively against *Rhizoctonia* spp., and were used to control rice sheath blight and wheat sharp eyespot in China [19,20,21,22]. Previously, our study demonstrated that both flutolanil and thifluzamide had a great efficiency on the *Rhizoctonia* isolates associated with sugar beet seedling damping-off, predominant AGs or subgroups of which were AG-4HGI, AG-4HGII, and AG-4HGIII [23,24]. Pencycuron is a phenylurea fungicide, which also has specific activity against *Rhizoctonia*, and is usually used to control rice sheath blight and potato stem canker or black scurf caused by *Rhizoctonia* [25,26]. However, there were no reports related to the effect of flutolanil, thifluzamide, and pencycuron on *Rhizoctonia* associated with sugar beet root and crown rot.

Mycoviruses are viruses that replicate in fungi and widespread in all major taxonomic groups of fungi [27,28]. To date, approximately 100 mycoviruses were found in *Rhizoctonia*, including members of established families accommodating double-stranded RNA (dsRNA), positive single-stranded RNA (+ssRNA), or negative single-stranded RNA (-ssRNA) viruses, together with members of proposed families and some unclassified RNA elements [29]. Mycoviruses were reported to be present in AG-1-IA [30,31], AG-2-2IIIB [32], AG-2-2IV [33,34], AG-2-2LP [35], AG-3 PT [36,37], AG-4HGI [38], AG-4HGIII [39], *R. oryzae-sativae* [40], *R. cerealis* [41], and *R. fumigata* [42], and the majority of identified mycoviruses were from *R. solani* AG-1-IA, the predominate pathogen causing rice sheath blight worldwide [29].

The metatranscriptomic and metagenomic sequencing technology is currently the most efficient approach for facilitating the identification of known and unreported mycoviruses in the target fungi [43,44]. In the last decade, many novel viruses were discovered using viral metagenomics [44]. However, only four studies concerning mycovirome of *Rhizoctonia*, which causes destructive economically important diseases on numerous crops, were reported. The mycoviral diversity of 84 isolates of *R. solani* (whose anastomosis groups or subgroups were unknown) collected from USA was investigated, and 27 mycoviruses with +ssRNA, -ssRNA, and dsRNA genomes were found [43]. Forty-seven partial or complete viral unique RNA-dependent RNA polymerase (RdRp) sequences with a high prevalence of -ssRNA viruses from eight strains of AG-2-2LP were gained using metatranscriptomic analysis [35]. A metatranscriptomic analysis of 43 isolates of *R. solani* AG-1-IA infecting rice that sampled in southern China was performed, and 10 mycovirus-related contigs composing five mycoviruses were obtained [45]. Recently, the diversity of putative mycoviruses containing +ssRNA, dsRNA, and -ssRNA genomes present in 66 strains of BNR (including AG-A, AG-Fa, AG-K, and AG-W) and 192 strains of MNR (including AG-1-IA, AG-2-1, AG-3 PT, AG-4HGI, AG-4HGII, AG-4HGIII, and AG-5) inciting potato stem canker or black scurf was studied using metatranscriptome sequencing, with four new parititviruses, 39 novel mitoviruses, and four new hypoviruses with nearly whole genome being detected in these 258 strains of BNR and MNR [46].

Until now, there are no detailed reports related to AGs or subgroups composition of *Rhizoctonia* isolates associated with sugar beet root and crown rot across China, let alone the diversity of mycoviruses present in these *Rhizoctonia* isolates as well as the sensitivity of them to the three fungicides (flutolanil, thifluzamide, and pencycuron). In the current study, the AGs or subgroups composition of *Rhizoctonia* isolates associated with sugar beet root and crown rot were characterized based on morphological traits and sequence analysis of internal transcribed spacer region of ribosomal DNA (rDNA-ITS) [10], the diversity of mycoviruses infecting these *Rhizoctonia* isolates were explored and analyzed by metatranscriptome sequencing [46], and the sensitivity of these *Rhizoctonia* isolates to the three fungicides (flutolanil, thifluzamide, and pencycuron) was detected using the mycelium growth inhibition method [23,24,47]. The results obtained in this study will be of great significance for understanding the AGs or subgroups composition of *Rhizoctonia* causing sugar beet rot and crown rot, revealing the diversity of mycoviruses discovered in *Rhizoctonia*, and screening efficient fungicides used for controlling diseases caused by *Rhizoctonia*.

## 2. Materials and Methods

### 2.1. Rhizoctonia Isolation and Identification

From 2009 to 2016, sugar beet roots with the symptoms of root and crown rot were collected from the sugar beet-growing regions across China, including Inner Mongolia and Xinjiang Uygur autonomous regions, Hebei, Heilongjiang, Gansu, Jilin, and Shanxi provinces, and Beijing municipality, which were used to isolate *Rhizoctonia*. Isolation, purification, and identification of *Rhizoctonia* were conducted according to the methods described in our previous study [10].

### 2.2. Pathogenicity Test

The pathogenicity of 61 representative *Rhizoctonia* isolates covering all the AGs or subgroups from different geographic origins was tested on eight-week-old sugar beet plants (cv. HI0305) under greenhouse conditions following the procedure described by Strausbaugh et al. [11] with a slight modification. The preparations of inoculum, planting soil, and sugar beet seedling were performed as previously described [10]. Two infested wheat seeds were placed at a depth of 10 mm into the soil next to the root of each eight-week-old sugar beet plant. Negative controls were inoculated with two un-infested autoclaved wheat seeds. The sugar beet plants were incubated in a greenhouse maintained at 25 to 27 °C with a 12 h photoperiod and watered whenever the surface soil appeared dry. Seven days later, all the plants including the control plants were harvested and used to assess disease incidence and disease index. Each treatment was conducted with three replicates, and ten sugar beet plants were used in each replicate. The experiment was arranged in a randomized block design and repeated twice.

Disease severity was rated using a 0–4 scale according to Harveson [48], which was described as follows: 0 = no disease, l = small, localized lesions with up to 25% of root surface affected, 2 = lesions coalescing with 26–50% of root affected, 3 = 51–75% of root covered with lesions but no internal discoloration, and 4 = more than 75% of beet surface covered with lesions and internal discoloration. The calculation of disease incidence and disease index was performed as described in our previous study [10]. All the sugar beet plants including the control plants were used to re-isolate *Rhizoctonia* and the resulting *Rhizoctonia* isolates were identified as the methods described in our previous study [10], fulfilling Koch’s postulates.

### 2.3. RNA Extraction, Metatranscriptomic Sequencing, and Sequence Analysis

For extracting total RNA, the 244 *Rhizoctonia* isolates identified above were cultured on potato dextrose agar (PDA) plates covered with cellophane film membranes (PDA-CF) at 25 °C in the dark for five days. Then, approximately 0.5 g of fresh mycelia were harvested and total RNAs were extracted using TRIzol Reagent (Invitrogen, Carlsbad, CA, USA) according to the manufacturer’s protocol. The extracted total RNA was further treated with RNase-Free DNase I to remove DNA contamination from RNA samples. The concentration and quality of RNA samples were measured using an ultramicro spectrophotometer (Nanodrop 2000, Thermo Scientific, Waltham, MA, USA), and the RNA integrity was further confirmed using 1.0% (*w*/*v*) gel electrophoresis. Finally, RNA samples were pooled by mixing 1 µg of RNA from each fungal sample to obtain one single sample with a final concentration (~200 ng/µL), which was sent to Shanghai Biotechnology Corporation (Shanghai, China) for metatranscriptomic sequencing using an Illumina X-TEN instrument with paired-end program.

TruSeq Stranded Total RNA LT Sample Prep Kit (Illumina, San Die-go, CA, USA) was used to establish sequencing library of the 244 *Rhizoctonia* isolates from rRNA-depleted total RNA. Library quality was checked using Qubit^®^ 2.0 Fluorometer (Invitrogen, Q32866) and Agilent Technologies 2100 Bioanalyzer (Agilent Technologies, Santa Clara, CA, USA). Clean data with high quality were obtained by filtering low quality reads (Q ≤ 20 bases accounted for more than 50% of the total bases), joint contamination reads (>5 bp) and reads containing more than 5% N in the original data using Trimmomatic. These clean reads were first matched against the genome sequences of *Rhizoctonia* using the Bowtie (1.0) software. The unmatched RNAs were next assembled into longer contiguous sequences (contigs) in the Velvet software. Subsequently, Cap3 and CD-Hit-Est (version 4.8.1) were used for splicing primary contigs and clustering them with 95% homologous data, respectively. Finally, the contigs obtained were employed for searching in non-redundant protein sequences (Nr) of GenBank database (http://www.ncbi.nlm.nih.gov/, accessed on 8 January 2021) in BLASTx program using the Diamond software (version 0.9.25) under default parameters with the exception of the e-value lower than 1 × 10^−5^ [49]. At last, the unmatched contigs with homologous to viral amino acid sequences, and with size of nucleotides over 1.0 kb or encoding a protein of at least 150 amino acids (aa), were regarded as potential viral sequences [50,51].

### 2.4. Open Reading Frame (ORF) Prediction and Phylogenetic Analysis

Nucleotide sequence of each contig was first analyzed for ORF prediction, which was performed using the ORF finder tool from NCBI (https://www.ncbi.nlm.nih.gov/orffinder/, accessed on 8 January 2021). Except for the contigs closely related to mitoviruses generally hosted in the mitochondria, which were analyzed using the “mold, protozoan and coelenterate mitochondrial” genetic code, predictions of the contigs related to other mycoviruses were conducted using the “standard” genetic code. The sequences which could encode an ORF that begins at the start codon and ends at the termination codon, were predicted to contain a complete ORF. Then, Conserved Domain Database (CDD) (with e-value of 0.01) (http://www.ncbi.nlm.nih.gov/Structure/cdd/wrpsb.cgi, accessed on 8 January 2021), Protein Family (Pfam) database (http://pfam.sanger.ac.uk/, accessed on 8 January 2021), and PROSITE database (http://www.expasy.ch/, accessed on 8 January 2021) were used to find conserved motifs of amino acid (aa) sequences of putative mycoviruses [37,46,52]. CLUSTAL_X was used to perform sequence alignments [53]. For phylogenetic analysis, the mycoviruses with complete RdRp domains were aligned with MUSCLE implemented in MEGA 6, and maximum likelihood (ML) trees were constructed in Jones–Taylor–Thornton (JTT) model with 1000 bootstrap replicates [54]. The reference sequences used in the phylogenetic tree were retrieved from GenBank.

### 2.5. Confirmation of Identified Mycoviruses in Rhizoctonia Populations

In order to confirm the potential viral sequences from metatranscriptomic sequencing and distribution of each mycovirus among the tested *Rhizoctonia* populations, the reverse transcription-polymerase chain reaction (RT-PCR) was conducted. The viral-specific primers (Table S1) were designed based on the virus sequences from metatranscriptomic sequencing, and the complementary DNA (cDNA) was synthesized using the High-Capacity cDNA Reverse Transcription Kit with RNase inhibitor (TaKaRa) from the total RNA that was extracted from the 244 *Rhizoctonia* isolates characterized. The RT-PCR was performed with a 25 µL PCR mixture consisted of 8.5 µL ddH_2_O, 12.5 µL 2 × *Pfu* Master Mix [PC1102 (Aidlab Biotechnology, Beijing, China), containing 0.05 U·µL^−1^ *Pfu* DNA polymerase, 400 μM dNTP, and 4 mM Mg^2+^], 1 µL each of viral-specific primers (10 µM), and 2 µL cDNA. The PCR products were verified using 1% agarose gel electrophoresis, and the amplified sequence with right size was sent to Beijing Tianyihuiyuan Co., Ltd. (Beijing, China) for sequencing. The amplification for each potential viral sequence was conducted three times and the amplification products of these three repetitions were sequenced separately.

### 2.6. Virus Name

The name of a novel putative mycovirus identified for the first time in this study is named according to previous references [46,55], which consists of three parts: (1) the first part is the source of the virus; (2) the second part shows the virus taxonomical group; and (3) the third part is a progressive number. For example, “Rhizoctonia solani [part 1] mitovirus [part 2] 41 [part 3]” presents a new mitovirus and the forty-first mitovirus found in *R. solani*. The threshold for identification of a new mycovirus was based on the ICTV report, which declared the species demarcation criteria of mycoviruses from each mycovirus family. For example, when the putative RdRp aa identity of a mitovirus was lower than 90%, the mitovirus was regarded as a new species in the family *Mitoviridae*. When a mycovirus was reported previously and identified also in this study, “strain beet” was labeled to indicate its host source in this study. For example, “Rhizoctonia solani partitivirus 2 strain beet” presents a strain of Rhizoctonia solani partitivirus 2 reported previously [56] and is identified from a strain of *R. solani* that isolated from sugar beet roots with the symptoms of root and crown rot in this study.

### 2.7. Sensitivity of Rhizoctonia Isolates to Flutolanil, Thifluzamide, and Pencycuron In Vitro

The sensitivity of all the 244 identified *Rhizoctonia* isolates to flutolanil, thifluzamide, and pencycuron were evaluated in vitro. Flutolanil [Active ingredient (AI): 99.50%], thifluzamide (AI: 96.00%), and pencycuron (AI: 99.50%) were provided by the Institute for the Control of Agrochemicals, Ministry of Agriculture and Rural Affairs (ICAMOARA), and dissolved in analytically pure methanol at 10.00 mg·mL^−1^ to make a stock solution. Then, the stock solutions of 10.00 mg·mL^−1^ of flutolanil, thifluzamide, and pencycuron were diluted with analytically pure methanol to obtain the gradient concentrations of 0.05, 0.10, 0.20, 0.40, and 0.80 mg·mL^−1^, 0.01, 0.02, 0.04, 0.08, and 0.16 mg·mL^−1^, and 0.005, 0.01, 0.02, 0.04, and 0.08 mg·mL^−1^, respectively. Finally, by adding appropriate volumes of each gradient concentration into the autoclaved PDA culture media prior to solidification (approximately 45 °C), the PDA culture media was amended with flutolanil, thifluzamide, and pencycuron to obtain final concentrations of 0.05, 0.10, 0.20, 0.40, and 0.80 μg·mL^−1^, 0.01, 0.02, 0.04, 0.08, and 0.16 μg·mL^−1^, and 0.005, 0.01, 0.02, 0.04, and 0.08 μg·mL^−1^, respectively, for testing its ability to inhibit the mycelial growth of the *Rhizoctonia* isolates. An equivalent amount of analytically pure methanol was added into non-amended PDA culture media, which was used as control. Isolates that showed an EC_50_ value ≥ 0.8 μg·mL^−1^, as was the case for pencycuron, were tested again using fungicide concentrations of 1.60, 3.20, 6.40, 15.00, and 30.00 μg·mL^−1^. The incubation of fungi and calculation of median effective concentration (EC_50_) values were conducted as described in our previous study [23,24].

### 2.8. Statistical Analysis

The statistical significance of disease incidence and disease index on sugar beet plants incited by the testing AGs or subgroups of *Rhizoctonia* and EC_50_ values of flutolanil, thifluzamide, and pencycuron to the testing AGs or subgroups of *Rhizoctonia* were analyzed with SPSS (Statistical Product and Service Solutions) software (version 20.0). Homogeneity of variance was assessed using Levene’s test. As variances and sample size were unequal, differences among different AGs or subgroups were tested via the non-parametric tests of Kruskal–Wallis H. The results were further examined by one-way analysis of variance (ANOVA) with Dunnett’s T3 tests (*p* = 0.05). Spearman’s rank correlation coefficients were used to determine whether logarithmic EC_50_ values between two tested fungicides were correlated with each other.

## 3. Results

### 3.1. AGs or Subgroups Determination of Rhizoctonia

In total, 244 *Rhizoctonia* isolates were successfully recovered from the diseased sugar beet roots with the symptoms of root and crown rot collected from Xinjiang Uygur and Inner Mongolia autonomous regions, Hebei, Heilongjiang, Gansu, Jilin, and Shanxi provinces, and Beijing municipality across China (Table S2) and identified to be AG-A, AG-K, AG-2-2IIIB, AG-2-2IV, AG-3 PT, AG-4HGI, AG-4HGII, and AG-4HGIII. Among the eight AGs or subgroups, the most predominant AGs or subgroups were AG-4HGI (108 isolates, 44.26%) and AG-2-2IIIB (107 isolates, 43.85%), followed by AG-4HGII (18 isolates, 7.38%), AG-A (five isolates, 2.05%), AG-K (two isolates, 0.82%), AG-2-2IV (two isolates, 0.82%), AG-3 PT (one isolate, 0.41%), and AG-4HGIII (one isolate, 0.41%) (Table 1).

**Figure 1:**
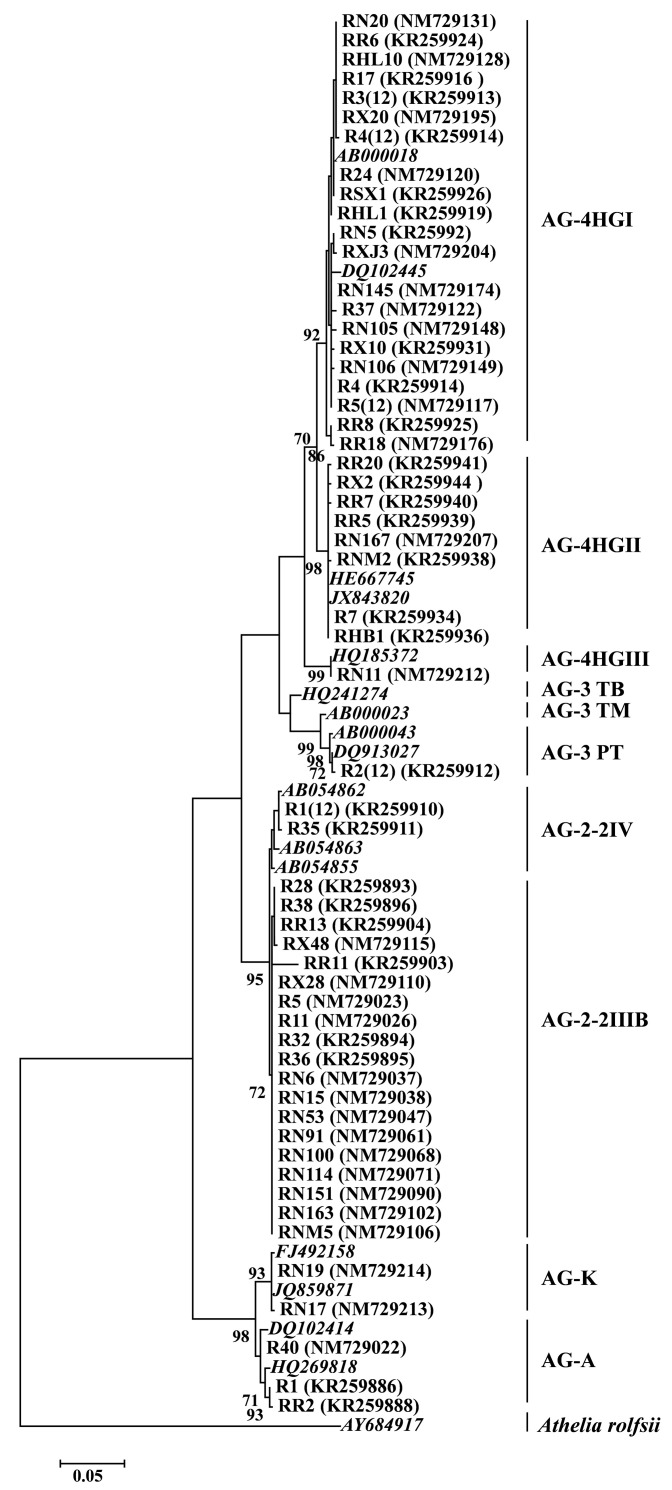
Phylogenetic tree constructed based on the sequences of internal transcribed spacer region of ribosomal DNA (rDNA-ITS) of 57 representative *Rhizoctonia* isolates in this study and 17 reference strains of *Rhizoctonia* retrieved from GenBank using neighbor-joining (NJ) method. Bootstrap values (1000 replicates) greater than 70 are shown above the branches. Scale bar represents a genetic distance of 0.05 for horizontal branch length. The sequence of rDNA-ITS of *Athelia rolfsii* FSR-052 (GenBank accession number AY684917) was used as outgroup.

Cluster analysis was performed using the sequences of rDNA-ITS of eight AGs or subgroups in this study and the reference AGs or subgroups retrieved from GenBank. Two distinct clades were mainly divided in the phylogenetic tree (Figure 1); one clade was composed of the BNR isolates belonging to AG-A and AG-K, and the other clade consisted of the MNR isolates belonging to AG-2, AG-3, and AG-4. Moreover, the *Rhizoctonia* isolates belonging to AG-2 and AG-4 in this study were further divided into two sub-clades (AG-2-2IIIB and AG-2-2IV) and three sub-clades (AG-4HGI, AG-4HGII, and AG-4HGIII), respectively.

### 3.2. Pathogenicity on Sugar Beet

The results of pathogenicity test indicated that except for *R. solani* AG-3 PT which could only form sclerotia on the surface of sugar beet roots, the remaining 60 tested *Rhizoctonia* isolates could cause mild or moderate root and crown rot symptoms on sugar beet roots, with brown and dry lesions on sugar beet roots or crowns. The disease incidence and the disease index of the sugar beet roots caused by the tested 60 *Rhizoctonia* isolates ranged from 29.10% to 100.00% and from 16.82 to 73.85, respectively (Table 2). Collectively, the BNR isolates (whose average disease index ranged from 16.82 to 19.98) were less aggressive than the MNR isolates (whose average disease index ranged from 61.57 to 73.85). No significant differences in disease index were detected among isolates representing the four AGs or subgroups of *Rhizoctonia*, namely AG-2-2IIIB, AG-2-2IV, AG-4HGI, and AG-4HGII, which were the most aggressive on sugar beet plants.

*Rhizoctonia* isolates were consistently re-isolated from symptomatic sugar beet plants which were inoculated with *Rhizoctonia*, and their identities were confirmed as the methods described above, fulfilling Koch’s postulates, whereas no *Rhizoctonia* isolates were re-isolated from the control plants which were asymptomatic.

### 3.3. Mycovirus Diversity in Rhizoctonia Isolates

A total of 67,270,624 raw reads yielding 10.09 GB data were obtained from the 244 *Rhizoctonia* isolates by metatranscriptome sequencing on an Illumina HiSeq 2500 platform, and were deposited in Sequence Read Archive (SRA) on the NCBI server (BioProject PRJNA766298). After filtering, the resulting clean reads were *de novo* assembled into 66,082 contigs with length >200 nt using CLC Genomics Workbench version 6.0.4 software. As a result of sequence analysis, 106 contigs with nearly complete or partial genomic sequences of viruses were obtained and composed 105 putative mono- or bi-segmented mycoviruses, more than half (56.19%) of which exhibited an amino acid (aa) identity less than 60% with the counterpart of their closest relatives (Table S3).

**Figure 2:**
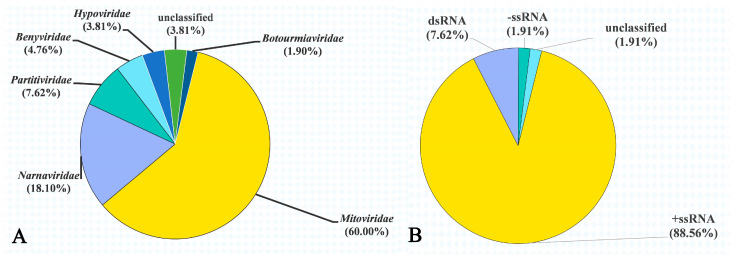
The diversity of mycoviruses present in 244 *Rhizoctonia* isolates associated with sugar beet root and crown rot. (**A**) Viral families identified in this study. Percentage in parentheses indicates the proportion of each virus family. (**B**) Nucleic acid types of mycoviruses identified in this study. Percentage in parentheses indicates the proportion of each type of nucleic acid.

Except for four unclassified mycoviruses (3.81%), the remaining 101 putative viral genomes showed affinity with six distinct families, namely *Mitoviridae* (63 putative viruses, 60.00%), *Narnaviridae* (19 putative viruses, 18.10%), *Partitiviridae* (eight putative viruses, 7.62%), *Benyviridae* (five putative viruses, 4.76), *Hypoviridae* (four putative viruses, 3.81%*)*, and *Botourmiaviridae* (two putative viruses, 1.90%) (Figure 2A). Moreover, the genomes of 105 putative mycoviruses contained three nucleic acid types, including +ssRNA (*Mitoviridae*, *Narnaviridae*, *Benyviridae*, *Hypoviridae, Botourmiaviridae,* and two unclassified mycoviruses), dsRNA (*Partitiviridae*), and –ssRNA (two unclassified mycoviruses), with +ssRNA (accounting for 88.57% of the total viruses) being the most predominant (Figure 2B).

Each of the tested 244 *Rhizoctonia* isolates carried at least one mycovirus. Among the 244 isolates, 54, 39, 31, 24, 11, 7, 5, 4, and 2 isolates were infected simultaneously by two, three, four, five, six, seven, eight, nine, and ten mycoviruses, respectively. Four isolates, RN136, RN137, RN138, and RN141, even carried 13, 18, 17, and 20 mycoviruses, respectively (Table S4). In addition, RsNV16 and RsPV2B were confirmed to be present in all the tested 244 *Rhizoctonia* isolates and 37 of the tested 244 *Rhizoctonia* isolates, respectively (Table S3).

### 3.4. Sequences Related to Putative Members of the Family Mitoviridae

**Figure 3:**
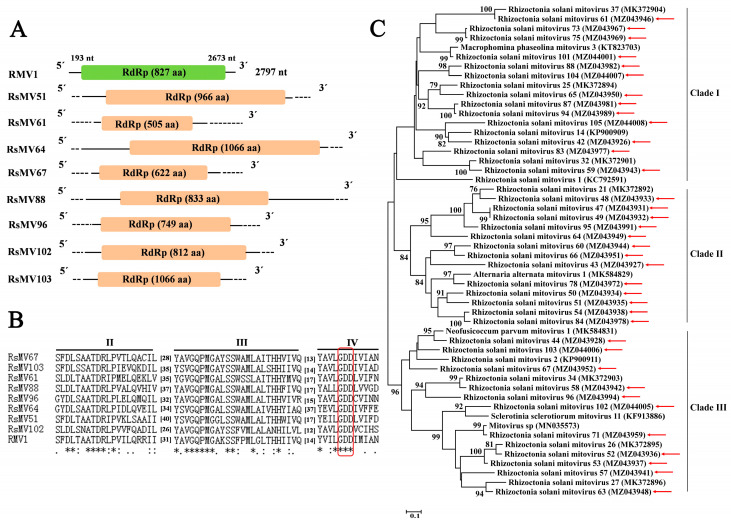
Genome organization, multiple alignment of amino acid (aa) sequence of RNA-dependent RNA polymerase (RdRp), and phylogenetic analysis of mitoviruses found in 244 *Rhizoctonia* isolates associated with sugar beet root and crown rot. (**A**) Schematic diagrams of genome organization of eight representative mitoviruses identified in this study and one reference virus, Rhizoctonia mitovirus 1 (RMV1, GenBank accession number KC792591). The colored boxes indicate hypothetical ORFs. Dotted lines represent undetermined untranslated regions (UTRs). (**B**) Multiple alignment of aa sequence of RdRp of eight representative mitoviruses identified in this study and one reference mitovirus, Rhizoctonia mitovirus 1 (RMV1, GenBank accession number KC792591). Three conserved motifs (motif II to IV) are indicated. The highly conserved GDD tripeptide present in mitoviruses is indicated by red boxes. “*” indicates identical aa, “:” indicates high chemically similar aa, and “.” indicates low chemically similar aa. (**C**) Phylogenetic tree constructed based on amino acid (aa) sequence of RNA-dependent RNA polymerase (RdRp) of 37 mitoviruses reported in this study and 15 reference viruses using the maximum-likelihood (ML) method in Jones–Taylor–Thornton (JTT) model with 1000 bootstrap replicates. Mycoviruses identified in this study are indicated by red arrows.

Sixty-three contigs (the length of nucleotide ranging from 1035 nt to 4397 nt) related to mycoviruses belonging to the family *Mitoviridae* were found in the 244 *Rhizoctonia* isolates, whose aa sequence identities with those of mycoviruses reported previously in the NCBI Nr database ranged from 39.83% to 89.66%, which were considered as novel viral species and named Rhizoctonia solani mitovirus 41 to 78, 80 to 96, and 98 to 105 (RsMV41–78, RsMV80–96, and RsMV98–105; Table S3). Among the sixty-three contigs, thirty-seven contigs related to mycoviruses were predicted to contain a complete ORF encoding RdRp, with the length of aa sequence ranging from 502 aa to 1066 aa. Contig ID, size, mycovirus name, best match, identity, query cover, E-value, and accession number were listed in Table 3. Eight contigs (Table 3) were chosen to carry out genome organization and multiple alignment. Based on mitochondrial codon usage, all the eight contigs related to mitoviruses contained an ORF encoding RdRp (Figure 3A). The results of multiple alignment indicated that three conserved motifs (motif II to motif IV) containing the GDD tripeptide in motif IV (characteristics of RdRp in mitoviruses) were found in the domain of RdRp in these eight viruses (Figure 3B).

The results of phylogenetic tree constructed based on the aa sequence of RdRps of the 37 putative mitoviruses identified in this study and 15 reference viruses belonging to the family *Mitoviridae* retrieved from NCBI database showed that the 37 putative mitoviruses belonged to the family *Mitoviridae* and were divided into three clades (clade I, II, and III) (Figure 3C).

### 3.5. Sequences Related to Putative Members of the Family Narnaviridae

**Figure 4:**
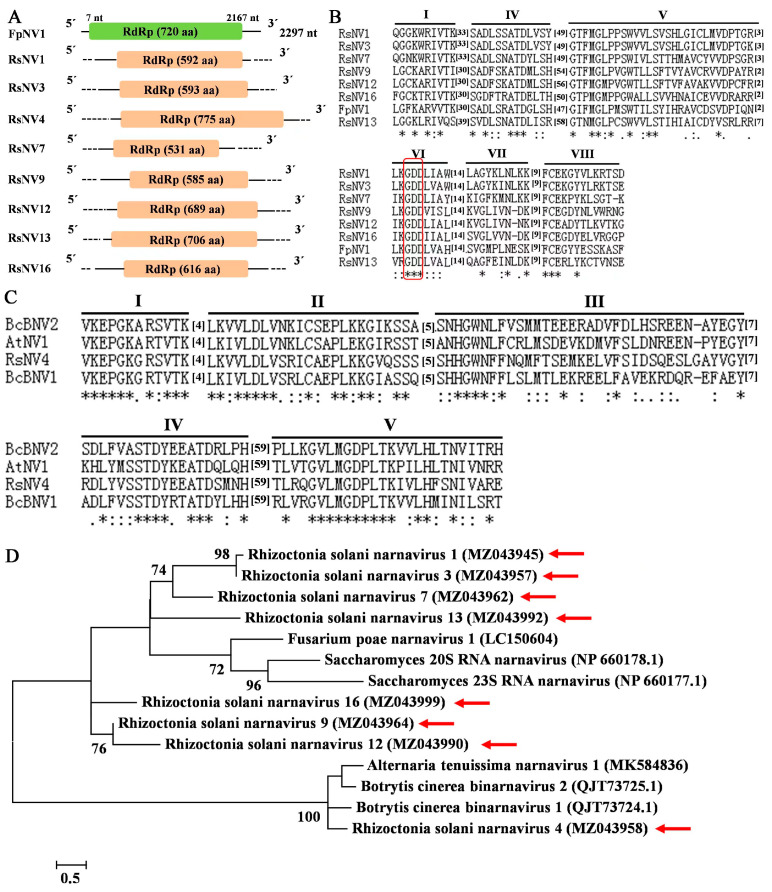
Genome organization, multiple alignment of amino acid (aa) sequence of RNA-dependent RNA polymerase (RdRp), and phylogenetic analysis of narnaviruses found in 244 *Rhizoctonia* isolates associated with sugar beet root and crown rot. (**A**) Schematic diagrams of genome organization of eight narnaviruses identified in this study and one reference virus, Fusarium poae narnavirus 1 (FpNV1, GenBank accession number LC150604). The colored boxes indicate hypothetical ORFs. Dotted lines represent undetermined untranslated regions (UTRs). (**B**) Multiple alignment of aa sequence of RdRp of seven narnaviruses identified in this study and one reference narnavirus, Fusarium poae narnavirus 1 (FpNV1, GenBank accession number LC150604). Six conserved motifs (motif I, IV to VIII) are indicated. The highly conserved GDD tripeptide present in narnaviruses is indicated by red boxes. “*” indicates identical aa, “:” indicates high chemically similar aa, and “.” indicates low chemically similar aa. (**C**) Multiple alignment of aa sequence of RdRp of Rhizoctonia solani narnavirus 4 (RsNV4) identified in this study and three reference viruses, namely Alternaria tenuisimma narnavirus 1 (AtNV1, GenBank accession number MK584836), Botrytis cinerea binarnavirus 1 (BcBNV1, GenBank accession number QJT73724.1), and Botrytis cinerea binarnavirus 2 (BcBNV2, GenBank accession number QJT73725.1). Five conserved motifs (motif I to V) are shown. “*” indicates identical aa, “:” indicates high chemically similar aa, and “.” indicates low chemically similar aa. (**D**) Phylogenetic tree constructed based on amino acid (aa) sequence of RNA-dependent RNA polymerase (RdRp) of eight narnaviruses reported in this study and six reference viruses using the ML method in Jones–Taylor–Thornton (JTT) model with 1000 bootstrap replicates. Mycoviruses identified in this study are indicated by red arrows.

Nineteen contigs related to mycoviruses in the family *Narnaviridae* were discovered in the 244 *Rhizoctonia* isolates, with the length of nucleotide sequence ranging from 1031 nt to 2408 nt, whose aa sequence identities with the reported viruses previously in the NCBI Nr database ranged from 27.97% to 58.50%. According to the classification criteria of the family *Narnaviridae* provided by International Committee on Taxonomy of Viruses (ICTV, https://ictv.global/report/chapter/narnaviridae/narnaviridae, accessed on 8 January 2021), these nineteen mycoviruses were considered as new viral species and named Rhizoctonia solani narnavirus 1 to 19 (RsNV1–19; Table S3). Among the 19 contigs, eight contigs (Contig 1113, Contig 8527, Contig 8560, Contig 11100, Contig 19187, First_Contig 917, First_Contig 1098, and First_Contig 5037) composing the eight putative mycoviruses, RsNV1, RsNV3, RsNV4, RsNV7, RsNV9, RsNV12, RsNV13, and RsNV16, respectively (Table 4), were predicted to have a complete ORF, with the length of aa sequence ranging from 531 aa to 775 aa, which were chosen to conduct genome organization (Figure 4A) and multiple alignment (Figure 4B,C). Contig ID, size, mycovirus name, best match, identity, query cover, E-value, and accession number are listed in Table 4. The results of multiple alignment revealed that the seven narnaviruses, namely RsNV1, RsNV3, RsNV7, RsNV9, RsNV12, RsNV13, and RsNV16, had the closest relationship to Fusarium poae narnairus 1 (FpNV1, GenBank accession number LC150604) and contained six conserved motifs (motif I, and motif IV to VIII) with a GDD tripeptide in motif VI (Figure 4B); however, RsNV4 was the most closely related to Alternaria tenuisimma narnavirus 1 (AtNV1, GenBank accession number MK584836), which contained five conserved motifs (motif I to motif V) without a GDD tripeptide (Figure 4C).

The results of phylogenetic tree constructed based on the aa sequence of RdRps of eight putative narnaviruses identified in this study and six reference viruses belonging to the family *Narnaviridae* retrieved from NCBI database showed that the eight putative narnaviruses were divided into two clades (Figure 4D). RsNV 1, 3, 7, 9, 12, 13, and 16 were clustered together with FpNV1, *Saccharomyces 20S RNA narnavirus* (ScNV-20S, GenBank accession number NP660178.1), and *Saccharomyces 23S RNA narnavirus* (ScNV-23S, GenBank accession number NP660177.1) (Figure 4D), while RsNV4 was clustered together with AtNV1, Botrytis cinerea binarnavirus 1 (BcBNV1, GenBank accession number QJT73724.1), and Botrytis cinerea binarnavirus 2 (BcBNV2, GenBank accession number QJT73725.1), the latter two of which was suggested to the members of a new proposed family Binarnaviridae [51] (Figure 4D).

### 3.6. Sequences Related to Putative Members of the Family Partitiviridae

Nine contigs composing eight mycoviruses which belonged to the family *Partitiviridae* were found in the 244 *Rhizoctonia* isolates, with the length of nucleotide sequence ranging from 930 nt to 2011 nt (Table S3). The aa sequences of coat protein (CP, Contig 2073) and RdRp (Contig 3529) of Rhizoctonia solani partitivirus 2 strain beet (RsPV2B) had identities of 99.39% and 99.52% with those of Rhizoctonia solani partitivirus (RsPV2) [55], respectively. With the exception of RsPV2B, seven other mycoviruses identified in this study had low aa sequence identities (47.52%–67.68%) with CP or RdRp domain of mycoviruses reported previously in the NCBI Nr databases, which were considered as new viral species and, thus, named Rhizoctonia solani partitivirus 15–21 (RsPV15–21).

**Figure 5:**
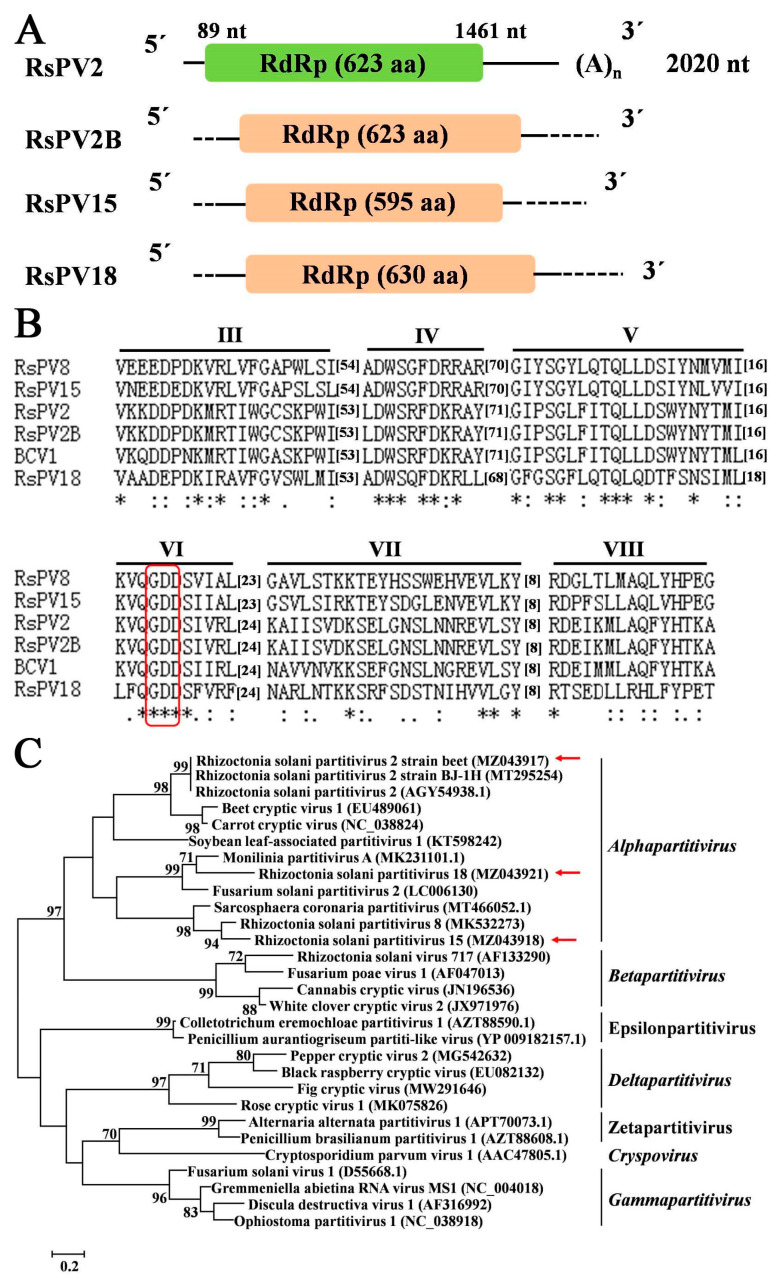
Genome organization, multiple alignment of amino acid (aa) sequence of RNA-dependent RNA polymerase (RdRp), and phylogenetic analysis of partitiviruses found in 244 *Rhizoctonia* isolates associated with sugar beet root and crown rot. (**A**): Schematic diagrams of genome organization of three partitiviruses identified in this study and one reference partitivirus, Rhizoctonia solani partitivirus 2 (RsPV2, GenBank accession number AGY54938.1). (**B**) Multiple alignment of aa sequence of RdRp of three partitiviruses identified in this study and three reference viruses, namely RsPV2, Rhizoctonia solani partitivirus 2 strain BJ-IH (RsPV2-BJ, GenBank accession number MT295254), and Beet cryptic virus1 (BCV1, GenBank accession number EU489061). The highly conserved GDD tripeptide present in partitiviruses is indicated by red boxes. “*” indicates identical aa, “:” indicates high chemically similar aa, and “.” indicates low chemically similar aa. (**C**) Phylogenetic tree constructed based on the aa sequences of RdRp of three partitiviruses identified in this study and 26 reference viruses using the ML method in Jones–Taylor–Thornton (JTT) model with 1000 bootstrap replicates. Mycoviruses identified in this study are indicated by red arrows.

Among the nine contigs, three contigs (Contig 3073, Contig 3529, and Contig 14630) composing the three putative mycoviruses, RsPV15, RsPV2B, and RsPV18, respectively, were predicted to have a complete ORF encoding RdRp, with the length of aa sequence ranging from 595 aa to 630 aa, which were chosen to perform genome organization (Figure 5A), multiple alignment (Figure 5B), and phylogenetic analysis (Figure 5C). Contig ID, size, mycovirus name, best match, identity, query cover, E-value, and accession number were listed in Table 5. Multiple alignment of the three partitiviruses (RsPV15, RsPV2B, and RsPV18) and three reference viruses revealed that five conserved motifs (motif III to motif VII) containing the GDD tripeptide were found to be present in RdRp domain of these six viruses (Figure 5B). Results of the phylogenetic tree constructed based on the RdRp aa sequence of these three partitiviruses in this study and representative members from five genera (*Alphapartitivirus*, *Betapartitivirus*, *Gammapartitivirus, Deltapartitivirus*, and *Cryspovirus*) and two proposed genera (Epsilonpartitivirus and Zetapartitivirus) within the family *Partitiviridae* indicated that the three mycoviruses (RsPV15, RsPV2B, and RsPV18) belonged to the genus *Alphapartitivirus* (Figure 5C); moreover, RsPV2B was clustered together with RsPV2 and Rhizoctonia solani partitivirus strain BJ-1H (RsPV2-BJ) [38] (Figure 5C).

### 3.7. Sensitivity of 244 Rhizoctonia Isolates to Flutolanil, Thifluzamide, and Pencycuron

The sensitivity of 244 *Rhizoctonia* isolates belonging to eight AGs or subgroups to flutolanil, thifluzamide, and pencycuron was evaluated (Table 6, *p* = 0.05). All the tested 244 *Rhizoctonia* isolates were highly sensitive to flutolanil and thifluzamide, with an average EC_50_ value of 0.3199 ± 0.0149 μg·mL^−1^ and 0.1081 ± 0.0044 μg·mL^−1^, respectively (Table S4). *Rhizoctonia* isolates belonging to AG-2-2IIIB, the most prevalent AG associated with sugar beet root and crown rot, was significantly less sensitive to flutolanil and thifluzamide than *Rhizoctonia* isolates belonging to AG-4HGI (Table 6, *p* = 0.05). All the *Rhizoctonia* isolates belonging to AG-2-2IIIB, AG-2-2IV, AG-3 PT, and AG-4HGIII, 107 of 108 *Rhizoctonia* isolates belonging to AG-4HGI, and six of eighteen *Rhizoctonia* isolates belonging to AG-4HGII were sensitive to pencycuron, with the average EC_50_ value of 0.0339 ± 0.0012 μg·mL^−1^ (Table S4). However, *Rhizoctonia* isolates belonging to AG-A and AG-K, one of 108 *Rhizoctonia* isolates belonging to AG-4HGI (R4), and 12 of 18 *Rhizoctonia* isolates belonging to AG-4HGII presented reduced sensitivity to pencycuron, with the average EC_50_ values of 6.6728 ± 1.2863 μg·mL^−1^ (Table S4).

**Figure 6:**
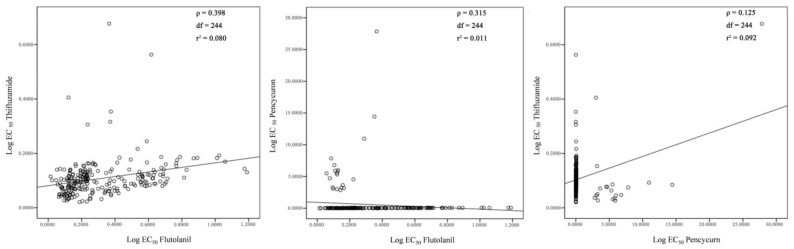
Cross-resistance of *Rhizoctonia* isolates to flutolanil, thifluzamide, and pencycuron was determined by a Spearman’s rank correlation analysis. Presented data represent the logarithmic values of the median effective concentrations (Log EC_50_) of the tested fungicides on mycelial growth of *Rhizoctonia* isolates.

In addition, the cross-resistance between the tested three fungicides was analyzed, and positive correlation between flutolanil and thifluzamide, flutolanil and pencycuron, and pencycuron and thifluzamide was low, with correlation index (*ρ*) of 0.398, 0.315, and 0.125, respectively (Figure 6).

## 4. Discussion

In this study, 244 *Rhizoctonia* isolates associated with sugar beet root and crown rot were identified and belonged to eight AGs or subgroups, including AG-A, AG-K, AG-2-2IIIB, AG-2-2IV, AG-3 PT, AG-4HGI, AG-4HGII, and AG-4HGIII. Among these eight AGs or subgroups, AG-4HGI (108 isolates, 44.26%) and AG-2-2IIIB (107 isolates, 43.85%) were the most prevalent. It is the first report that AG-A, AG-K, AG-4HGII, and AG-4HGIII can cause sugar beet root and crown rot in China. It was demonstrated that the eight AGs or subgroups mentioned above could cause sugar beet seedling damping-off in our previous study [10], inferring that effective management of seedling damping-off incited by these eight AGs or subgroups of *Rhizoctonia* could be beneficial to prevent sugar beet plants in the later growing season from infection of them. *R. solani* AG-2-2IIIB was proven to be the dominant AG associated with sugar beet root and crown rot in the present study, which was in accordance with the results reported in previous studies [5,6]. Generally, *R. solani* AG-4HGI was regarded as the dominant pathogen associated with sugar beet seedling damping-off [8,9,10], but the results in the current study confirmed that *R. solani* AG-4HGI was also the predominate AG causing sugar beet root and crown rot. In 2015, there was one report that sugar beet root and crown rot could be caused by *R. solani* AG-4HGI in China [13]. Interestingly, our previous study demonstrated that *R. solani* AG-3 TB could cause sugar beet seedling damping-off [10], which was reported as the causal agent of tobacco target leaf spot [57]. However, AG-3 PT, the predominate pathogen causing potato stem canker or black scurf [58], could not incite root and crown rot on sugar beet roots in this study, which only formed sclerotia on the surface of eight-week-old sugar beet roots.

To date, more than 100 mycoviruses were found in *Rhizoctonia*, most of which belong to the nine families, namely *Barnaviridae*, *Botourmiaviridae*, *Deltaflexiviridae*, *Endornaviridae*, *Hypoviridae*, *Megabirnaviridae*, *Mitoviridae*, *Partitiviridae*, and *Fusariviridae* [29,32,37]. There were only two publications which reported mycoviruses identified from *Rhizoctonia* isolates causing sugar beet root and crown rot; one publication was that a *R. solani* AG-2-2IV strain DC17 was infected by 17 viral species through deep sequencing analysis, and complete genome of Rhizoctonia solani flexivirus 1 (RsFV-1) was further sequenced and analyzed to confirm that it belonged to the order *Tymovirales* [33,34]; the other publication was that a new mitovirus, Rhizoctonia solani mitovirus 39 (RsMV-39), was isolated from *R. solani* AG-2-2IIIB strain RR17 in our previous study [32]. In the current study, 105 putative mycoviruses were found in 244 *Rhizoctonia* isolates; excluding four unclassified mycoviruses, the remaining 101 putative mycoviruses belonged to the six families, including *Benyviridae*, *Botourmiaviridae*, *Hypoviridae*, *Mitoviridae*, *Narnaviridae*, and *Partitiviridae*, with 63 mycoviruses (62.38%) being assigned to the family *Mitoviridae*, which was approximately consistent with the previous studies [33,35,43]. This was the first detailed record of the putative mycoviruses associated with *Rhizoctonia* causing sugar beet root and crown rot in China and perhaps worldwide using metatranscriptome sequencing.

*Mitoviridae* is a newly established family designated by the ICTV (2019) and comprises mitoviruses from plants and fungi, which typically replicate and persist in the mitochondrion of a host [59]. Viruses of the family *Mitoviridae* are known to be the simplest naked mycoviruses and exist as RNA–RdRp nucleoprotein complexes, whose genomes only encode an RdRp protein [28]. In the present study, 37 mitoviruses predicted to have a complete ORF were divided into three clades (clade I, clade II, and clade III) in the phylogenetic tree (Figure 3), whose aa sequence identities of RdRp with those of the corresponding mitoviruses in the NCBI Nr database ranged from 39.83% to 89.66%; moreover, the result of phylogenetic analysis is consistent with that of the previous reports [37,46,60,61].

Only two narnaviruses, namely *Saccharomyces 20S RNA narnavirus* (ScNV-20S) and *Saccharomyces 23S RNA narnavirus* (ScNV-23S) [62], were recognized by the ICTV. In the present study, 19 putative narnaviruses were obtained, eight of which were predicted to encode a complete ORF; furthermore, it was the first record of narnaviruses discovered in *Rhizoctonia*. It is noteworthy that RsNV4 recorded in this study as well as three reference viruses (AtNV1, BcBNV1, and BcBNV1) does not contain a typical “GDD” motif and is the same as the narnavius, Magnaporthe oryzae narnavirus virus 1 (MoNV1), isolated from *Magnaporthe oryzae* [63], which is highly conserved in almost all viral RdRps and was deduced to be part of the catalytic site of RdRp [64,65].

Partitiviruses, with bi-segmented genomes, have broad host ranges and are usually associated with latent infections in fungi, plants, and protozoa [27,66]. In general, five genera, namely *Alphapartitivirus*, *Betapartitivirus*, *Gammapartitivirus*, *Deltapartitivirus*, and *Cryspovirus*, were recognized in the family *Partitiviridae*. The members in each of these five genera of the family *Partitiviridae* are corresponding to typical hosts: alphapartitiviruses and betapartitiviruses infect plants and fungi, gammapartitiviruses and deltapartitiviruses only infect fungi and plants, respectively, and cryspoviruses only comprise viruses isolated from protozoa by now [67]. Recently, two new genera, “Epsilonpartitivirus” and “Zetapartitivirus”, were proposed to be established in the family *Partitiviridae* [68,69]. In this study, nine contigs related to partitiviruses were obtained, three of which could encode a complete RdRp and were clustered into the genus *Alphapartitivirus* (Figure 5).

*R. solani* AG-2-2IV isolate DC17 was reported to harbor 17 mycoviruses, including six mitoviruses, three Sclerotinia sclerotiorum RNA virus L-like viruses, two putative members of the order *Tymovirales*, one endornavirus, one partitivirus, one megabirnavirus, one Aspergillus foetidus slow virus 2-like virus, and one Rhizoctonia solani dsRNA virus 1-like virus [33]. Chen et al. [70] reported that at least three novel betapartitiviruses co-infected the phytopathogenic fungus *R. solani*. *R. solani* AG-3 PT RS002 isolate infecting potato harbored an endornavirus and a mitovirus [36,71]. Recently, we reported that six novel mycoviruses containing +ssRNA and dsRNA genomes co-infected a single strain of *R. solani* AG-3 PT [37]. In this study, each of the tested 244 *Rhizoctonia* isolates were determined to carry at least one mycovirus; 181 (74.18%) out of these 244 *Rhizoctonia* isolates were infected by at least two mycoviruses simultaneously; moreover, four isolates even carried more than ten mycoviruses.

Flutolanil and thifluzamide are SDHI fungicides, and have high efficiency on controlling diseases caused by *Rhizoctonia*. Flutolanil was reported to control rice sheath blight [20], wheat sharp eyespot [21], potato stem canker or black scurf [72], peanut stem rot [73], tall fescue brown patch [74], sugar beet seedling damping-off [23], and sugar beet root and crown rot [18]. Thifluzamide was recorded to control rice sheath blight, wheat sharp eyespot, and sugar beet seedling damping-off [19,22,24]. As a phenylurea fungicide, pencycuron had specific activity against *Rhizoctonia*, and was usually used to control seedling diseases of crops, such as rice sheath blight and potato stem canker or black scurf caused by *Rhizoctonia* [25,26]. In this study, all the 244 tested *Rhizoctonia* isolates were highly sensitive to flutolanil and thifluzamide; 224 *Rhizoctonia* isolates (including 107 isolates of AG-2-2IIIB, two isolates of AG-2-2IV, one isolate of AG-3 PT, 107 isolates of AG-4HGI, six isolates of AG-4HGII, and one isolate of AG-4HGIII) were sensitive to pencycuron, but the remaining 20 *Rhizoctonia* isolates (including seven isolates of AG-A and AG-K, one isolate of AG-4HGI, and 12 isolates of AG-4HGII) presented a reduced sensitivity to pencycuron, with EC_50_ of pencycuron on these 20 isolates being more than 2.00 μg·mL^−1^. It was previously reported that *R. solani* AG-2 and AG-3 were highly sensitive to pencycuron, while AG-4, AG-5, and AG-7 only presented moderate to low level of sensitivity to pencycuron [75,76]. *R. solani* AG-3, AG-4HGI, and AG-4HGII associated with potato black scarf disease were sensitive to pencycuron, with AG-3 being more sensitive than AG-4HGI and AG-4HGII [77]. Therefore, it was concluded that flutolanil and thifluzamide would be the suitable fungicides for controlling sugar beet root and crown rot caused by *Rhizoctonia* in China. Since different AGs presented different level of sensitivity to pencycuron and AGs composition in each producing region of sugar beet in China was various, AGs composition in a certain production region of sugar beet should be identified at first, when pencycuron was chosen to control root and crown rot caused by *Rhizoctonia* in this region. In addition, positive correlation between flutolanil and thifluzamide, flutolanil and pencycuron, and thifluzamide and pencycuron was low, which inferred that these three fungicides could be used for controlling sugar beet root and crown rot caused by *Rhizoctonia* with rotation or mixture.

Previous studies reported that mycoviruses could affect the sensitivity of their host fungi to fungicides. Niu et al. [78] found that the fungicide-resistant *Penicillium digitatum* strains HS-F6 and HS-E9 co-infected by Penicillium digitatum polymycovirus 1 and Penicillium digitatum Narna-like virus 1 exhibited obvious reduction in resistance to the demethylation inhibitor (DMI)-fungicide, prochloraz. Wang et al. [79] reported that when *P. crustosum* strain HS-CQ15 was infected by Penicillium crustosum chrysovirus 1, the resistance of it to prochloraz decreased. In our previous study, the sensitivity of *Alternaria alternata* strain SD-BZF-12 infected by Alternaria alternata chrysovirus 1-AT1 to difenoconazole and tebuconazole reduced [80]. Our recent study demonstrated that Alternaria alternata botybirnavirus 1-AT1 decreased the sensitivity of its host *A. tenuissima* strain TJ-NH-51S-4 to difenoconazole [81]. In the present study, each of the tested 244 *Rhizoctonia* isolates presented different sensitivity to the three fungicides (flutolanil, thifluzamide, and pencycuron). For example, among the 108 isolates of AG-4HGI, the EC_50_ of pencycuron on isolate R4 was 3.2941 μg·mL^−1^, while the EC_50_ of pencycuron on the remainder 107 isolates ranged from 0.0049 μg·mL^−1^ to 0.0581 μg·mL^−1^. Moreover, the number and species of mycoviruses associated with each of these 244 isolates were diverse. Collectively, it was inferred that the varied sensitivity of these isolates to fungicides might be related to the mycoviruses associated with them. In the future, whether mycoviruses could affect the sensitivity of their host fungi to fungicides or not needs to be further studied.

## Figures and Tables

**Table 1:** Geographic origin and number of *Rhizoctonia* isolates isolated from sugar beet roots with the symptoms of root and crown rot in China.

Geographic Origin	Number of Isolates in Different Anastomosis Groups (AGs) or Subgroups of *Rhizoctonia*							
	AG-A	AG-K	AG-2-2IIIB	AG-2-2IV	AG-3 PT	AG-4HGI	AG-4HGII	AG-4HGIII
Beijing municipality	0	0	0	1 (1) ^z^	0	0	0	0
Gansu province	0	0	0	0	0	1 (1)	1 (1)	0
Hebei province	0	0	0	0	0	0	4 (1)	0
Heilongjiang province	5 (5)	0	4 (3)	1 (1)	1 (1)	18 (5)	3 (2)	0
Inner Mongolia autonomous region	0	2 (2)	93 (12)	0	0	50 (7)	8 (3)	1 (1)
Jilin province	0	0	1 (1)	0	0	0	0	0
Shanxi province	0	0	2 (2)	0	0	7 (2)	0	0
Xinjiang Uygur autonomous region	0	0	7 (4)	0	0	32 (6)	1 (1)	0
Total	5 (5)	2 (2)	107 (22)	2 (2)	1 (1)	108 (21)	18 (8)	1 (1)
Ratio (%)	2.05	0.82	43.85	0.82	0.41	44.26	7.38	0.41

**Note:** ^z^ Number in parentheses was the number of corresponding AG or subgroup of *Rhizoctonia* isolates used for pathogenicity tests.

**Table 2:** Disease incidence and disease index on eight-week-old sugar beet plants caused by different anastomosis groups (AGs) or subgroups of *Rhizoctonia* isolates.

AGs or Subgroups of *Rhizoctonia*	Disease Incidence (%) ^y^	Disease Index ^y^
AG-A	29.10 ± 6.28 b	19.98 ± 3.87 b
AG-K	54.35 ± 24.55 b	16.82 ± 7.80 b
AG-2-2IIIB	80.83 ± 6.60 ab	61.57 ± 4.59 a
AG-2-2IV	95.70 ± 4.30 a	68.20 ± 18.20 a
AG-3 PT ^z^	0.00	0.00
AG-4HGI	96.69 ± 1.42 a	73.85 ± 3.39 a
AG-4HGII	98.12 ± 1.26 a	68.70 ± 10.29 a
AG-4HGIII ^z^	100.00	72.73

**Note:** ^z^ Only one isolate of *Rhizoctonia* was obtained in AG-3 PT or AG-4HGIII. ^y^ Letters following the disease incidence and the disease index in the same column indicate significantly different averages (*p* = 0.05) based on one-way analysis of variance (ANOVA) with Dunnett’s T3 tests.

**Table 3:** The information of thirty-seven mitoviruses with complete open reading frame (ORF) encoding RNA dependent-RNA polymerases (RdRp) found in *Rhizoctonia* isolates associated with sugar beet root and crown rot.

Contig ID	Size (Amino Acid)	Mycovirus Name	Best Match	Identity (Amino Acid)	Query Cover	E-Value	Accession Number
Contig 190	1001	Rhizoctonia solani mitovirus 42	Rhizoctonia solani mitovirus 14	57.91%	70%	0.0	MZ043926
Contig 195	879	Rhizoctonia solani mitovirus 43	Epicoccum nigrum mitovirus 1	53.20%	66%	0.0	MZ043927
Contig 249	697	Rhizoctonia solani mitovirus 44	Neofusicoccum parvum mitovirus 1	88.52%	78%	0.0	MZ043928
Contig 370	898	Rhizoctonia solani mitovirus 47	Macrophomina phaseolina mitovirus 3	41.43%	80%	0.0	MZ043931
Contig 466	1066	Rhizoctonia solani mitovirus 48	Rhizoctonia solani mitovirus 21	58.70%	70%	0.0	MZ043932
Contig 468	1041	Rhizoctonia solani mitovirus 49	Rhizoctonia solani mitovirus 21	76.89%	71%	0.0	MZ043933
Contig 508	967	Rhizoctonia solani mitovirus 50	Mitovirus sp.	42.71%	57%	2 × 10^−179^	MZ043934
Contig 509	966	Rhizoctonia solani mitovirus 51 ^z^	Mitovirus sp.	43.61%	57%	0.0	MZ043935
Contig 538	781	Rhizoctonia solani mitovirus 52	Rhizoctonia solani mitovirus 26	84.54%	89%	0.0	MZ043936
Contig 539	783	Rhizoctonia solani mitovirus 53	Rhizoctonia solani mitovirus 26	85.06%	77%	0.0	MZ043937
Contig 604	844	Rhizoctonia solani mitovirus 54	Mitovirus sp.	48.70%	51%	5 × 10^−168^	MZ043938
Contig 851	811	Rhizoctonia solani mitovirus 57	Rhizoctonia solani mitovirus 8	72.80%	81%	0.0	MZ043941
Contig 997	869	Rhizoctonia solani mitovirus 58	Rhizoctonia solani mitovirus 34	80.92%	76%	0.0	MZ043942
Contig 1038	887	Rhizoctonia solani mitovirus 59	Rhizoctonia solani mitovirus 32	70.90%	64%	0.0	MZ043943
Contig 1104	850	Rhizoctonia solani mitovirus 60	Epicoccum nigrum mitovirus 1	75.54%	70%	0.0	MZ043944
Contig 1212	505	Rhizoctonia solani mitovirus 61 ^z^	Rhizoctonia solani mitovirus 37	77.25%	83%	0.0	MZ043946
Contig 1830	855	Rhizoctonia solani mitovirus 63	Rhizoctonia solani mitovirus 27	77.09%	76%	0.0	MZ043948
Contig 2377	1066	Rhizoctonia solani mitovirus 64 ^z^	Rhizoctonia solani mitovirus 1	72.18%	35%	0.0	MZ043949
Contig 3007	835	Rhizoctonia solani mitovirus 65	Rhizoctonia solani mitovirus 25	62.19%	65%	0.0	MZ043950
Contig 3241	838	Rhizoctonia solani mitovirus 66	Epicoccum nigrum mitovirus 1	85.00%	71%	0.0	MZ043951
Contig 3312	622	Rhizoctonia solani mitovirus 67 ^z^	Neofusicoccum parvum mitovirus 1	46.21%	87%	0.0	MZ043952
Contig 10147	804	Rhizoctonia solani mitovirus 71	Mitovirus sp.	89.66%	77%	0.0	MZ043959
First_Contig 14	715	Rhizoctonia solani mitovirus 73	Macrophomina phaseolina mitovirus 3	42.95%	82%	5 × 10^−175^	MZ043967
First_Contig 20	715	Rhizoctonia solani mitovirus 75	Macrophomina phaseolina mitovirus 3	42.97%	80%	1 × 10^−169^	MZ043969
First_Contig 27	847	Rhizoctonia solani mitovirus 78	Alternaria alternata mitovirus 1	80.87%	71%	0.0	MZ043972
First_Contig 139	949	Rhizoctonia solani mitovirus 83	Rhizoctonia solani mitovirus 7	70.06%	72%	0.0	MZ043977
First_Contig 203	876	Rhizoctonia solani mitovirus 84	Epicoccum nigrum mitovirus 1	47.54%	57%	8 × 10^−161^	MZ043978
First_Contig 275	864	Rhizoctonia solani mitovirus 87	Rhizoctonia solani mitovirus 25	47.64%	77%	0.0	MZ043981
First_Contig 345	833	Rhizoctonia solani mitovirus 88 ^z^	Rhizoctonia solani mitovirus 33	62.34%	21%	2 × 10^−122^	MZ043982
First_Contig 740	730	Rhizoctonia solani mitovirus 94	Rhizoctonia solani mitovirus 25	45.35%	93%	0.0	MZ043989
First_Contig 919	809	Rhizoctonia solani mitovirus 95	Rhizoctonia solani mitovirus 21	55.36%	90%	0.0	MZ043991
First_Contig 1173	749	Rhizoctonia solani mitovirus 96 ^z^	Rhizoctonia solani mitovirus 11	41.36%	79%	2 × 10^−164^	MZ043994
Second_Contig 58	785	Rhizoctonia solani mitovirus 101	Macrophomina phaseolina mitovirus 3	86.19%	93%	0.0	MZ044001
Contig 11664	812	Rhizoctonia solani mitovirus 102 ^z^	Sclerotinia sclerotiorum mitovirus 11	74.05%	38%	0.0	MZ044005
Contig 163	731	Rhizoctonia solani mitovirus 103 ^z^	Rhizoctonia solani mitovirus 2	56.04%	75%	0.0	MZ044006
Contig 1141	803	Rhizoctonia solani mitovirus 104	Rhizoctonia solani mitovirus 25	48.38%	66%	3 × 10^−175^	MZ044007
Contig 1714	866	Rhizoctonia solani mitovirus 105	Rhizoctonia solani mitovirus 14	52.10%	69%	0.0	MZ044008

**Note:** ^‘z’^ indicated mycoviruses selected for genome organization and multiple alignment analyses.

**Table 4:** The information of eight narnaviruses with complete open reading frame (ORF) encoding RNA dependent-RNA polymerases (RdRp) found in *Rhizoctonia* isolates associated with sugar beet root and crown rot.

Contig ID	Size (Amino Acid)	Mycovirus Name	Best Match	Identity (Amino Acid)	Query Cover	E-Value	Accession Number
Contig 1113	592	Rhizoctonia solani narnavirus 1	Fusarium poae narnavirus 1	30.83%	45%	4 × 10^−27^	MZ043945
Contig 8527	593	Rhizoctonia solani narnavirus 3	Fusarium poae narnavirus 1	30.71%	44%	3 × 10^−28^	MZ043957
Contig 8560	775	Rhizoctonia solani narnavirus 4	Alternaria tenuissima narnavirus 1	45.55%	88%	0.0	MZ043958
Contig 11100	531	Rhizoctonia solani narnavirus 7	Fusarium poae narnavirus 1	31.72%	45%	1 × 10^−24^	MZ043962
Contig 19187	585	Rhizoctonia solani narnavirus 9	Fusarium poae narnavirus 1	28.98%	54%	8 × 10^−30^	MZ043964
First_Contig 917	689	Rhizoctonia solani narnavirus 12	Fusarium poae narnavirus 1	28.68%	60%	1 × 10^−36^	MZ043990
First_Contig 1098	706	Rhizoctonia solani narnavirus 13	Fusarium poae narnavirus 1	28.29%	64%	3 × 10^−18^	MZ043992
First_Contig 5037	616	Rhizoctonia solani narnavirus 16	Fusarium poae narnavirus 1	28.96%	55%	3 × 10^−34^	MZ043999

**Table 5:** The information of three partitiviruses with complete open reading frame (ORF) encoding RNA dependent-RNA polymerases (RdRp) found in *Rhizoctonia* isolates associated with sugar beet root and crown rot.

Contig ID	Size (Amino Acid)	Mycovirus Name	Best Match	Identity (Amino Acid)	Query Cover	E-Value	Accession Number
Contig 3529	623	Rhizoctonia solani partitivirus 2 strain beet	Rhizoctonia solani dsRNA virus 2	99.52%	95%	0.0	MZ043917
Contig 3073	595	Rhizoctonia solani partitivirus 15	Rhizoctonia solani partitivirus 8	67.68%	94%	0.0	MZ043918
Contig 14630	630	Rhizoctonia solani partitivirus 18	Fusarium solani partitivirus 2	47.52%	88%	0.0	MZ043921

**Table 6:** Median effective concentration (EC_50_, μg·mL^−1^) of flutolanil, thifluzamide, and pencycuron on different anastomosis groups (AGs) or subgroups of *Rhizoctonia* isolates associated with sugar beet root and crown rot.

AGs or Subgroups of *Rhizoctonia*	Number of Isolates	Mean of EC_50_ (μg·mL^−1^) of Fungicide on *Rhizoctonia* ^y^		
		Flutolanil	Thifluzamide	Pencycuron
AG-A	5	0.1568 ± 0.1235 b	0.2797 ± 0.2588 a	8.8470 ± 10.6742 b
AG-K	2	0.3201 ± 0.0440 ab	0.0889 ± 0.0055 c	12.6914 ± 2.4621 a
AG-2-2IIIB	107	0.5071 ± 0.2288 a	0.1200 ± 0.0714 b	0.0372 ± 0.0200 d
AG-2-2IV	2	0.3220 ± 0.0651 ab	0.0828 ± 0.0189 c	0.0124 ± 0.0061 d
AG-3 PT ^z^	1	0.2330	0.0979	0.0109
AG-4HGI	108	0.1754 ± 0.0833 b	0.0958 ± 0.0328 c	0.0623 ± 0.3142 d
AG-4HGII	17	0.1309 ± 0.0393 b	0.0728 ± 0.0402 c	3.5671 ± 2.7024 c
AG-4HGIII ^z^	1	0.1288	0.0476	0.0238

**Note:** ^z^ Only one isolate of *Rhizoctonia* was obtained in AG-3 PT or AG-4HGIII. ^y^ Letters following the mean of median effective concentration (EC_50_, μg·mL^−1^) in the same column indicate significantly different averages (*p* = 0.05) based on one-way analysis of variance (ANOVA) with Dunnett’s T3 tests.

## Data Availability

The sequences of internal transcribed spacer region of ribosomal DNA (rDNA-ITS) of 244 *Rhizoctonia* isolates reported in this study have been deposited in the GenBank database under accession numbers of KP259886, KP259888 to KP259897, KP259899 to KP259904, KP259906, KP259908, KP259910 to KP259916, KP259918 to KP259920, KP259922 to KP259932, KP259934 to KP259944, NM729020 to NM729029, and NM729031 to NM729214. The sequences of 105 putative mycoviruses reported in this study have been deposited in the GenBank database under accession numbers of MZ043901 to MZ043972, MZ043974 to MZ043994, MZ043996 to MZ44008. All raw data of RNA-seq are available at NCBI Sequence Read Archive (BioProject PRJNA766298).

## Supplementary Materials

The following are available online at https://www.mdpi.com/article/10.3390/jof9050545/s1: Table S1: The primer pairs used to verify the 105 putative mycoviruses identified in this study and to detect the distribution of them in the 244 tested *Rhizoctonia* isolates. Table S2: *Rhizoctonia* isolates recovered from sugar beet roots with the symptoms of root and crown rot in China from 2009 to 2016. Table S3: Assembled sequences of mycoviruses found in *Rhizoctonia* isolates associated with sugar beet root and crown rot and their amino acid (aa) identities to those of viruses described previously. Table S4: Mycoviruses associated with 244 *Rhizoctonia* isolates recovered from sugar beet roots with the symptoms of root and crown rot in China from 2009 to 2016 and the median effective concentration (EC_50_) of flutolanil, thifluzamide, and pencycuron to these *Rhizoctonia* isolates.
