# Supplementary material for: Anastomosis Groups and Mycovirome of Rhizoctonia Isolates Causing Sugar Beet Root and Crown Rot and Their Sensitivity to Flutolanil, Thifluzamide, and Pencycuron
Source: J Fungi (Basel). 2023 May 9;9(5):545. doi: 10.3390/jof9050545 (PMC10219533; doi:10.3390/jof9050545)
Supplement: Supplementary file 1 [file jof-09-00545-s001.zip › Table S1.pdf]

Table S1. The primer pairs used to verify the 105 putative mycoviruses identified in this study and to detect the distribution of them in the 244 tested *Rhizoctonia* isolates.

**Name of mycovirus**	**Sequence of forward primer (5´-3´)**	**Sequence of reward primer (5´-3´)**
Rhizoctonia solani beny-like virus 2	TACTTACGCACGGGTTGT	CACTGGGTTAGGCAGAAAT
Rhizoctonia solani beny-like virus 3	TAGTCTGCCATCGGTGCG	GGGTTGGGCAAGGTTCTG
Rhizoctonia solani beny-like virus 4	TAAAGAGTTCGGCAGGTC	ATGAAAGCGTGGAAGAGG
Rhizoctonia solani beny-like virus 5	CGTTTCTTTCTCGGGTCA	CGTCGTGGAAGTTGCTAG
Rhizoctonia solani beny-like virus 6	ATGAGCCGATGGGTAGTTC	AAGGTTTGAGGGTGGGTA
Rhizoctonia solani hypovirus 5	CGCTCCTCACGGCTCTAT	GCAATGTGCCTGCCAGTA
Rhizoctonia solani hypovirus 6	CCGTTTGCCACTGCTTCC	CTCGCCATCTCGCATCCT
Rhizoctonia solani hypovirus 7	GAACTCGAAAGCGTAAATCA	CCTATACTGGCTCCAAATGC
Rhizoctonia solani hypovirus 8	TTACCTTCCTTGCGGCTCTT	TGTTCTTGGCACGGCTGA
Rhizoctonia solani mitovirus 41	AACCCAAGACTTTGAGGATC	ACGAGGATAAAGCACCCA
Rhizoctonia solani mitovirus 42	CAACTTAGACCTGACCCT	TAACTGAATGGCTTTGAC
Rhizoctonia solani mitovirus 43	GCTATTGTTAGCGGTATTT	ATTGCGTCCTTATTCAGA
Rhizoctonia solani mitovirus 44	GGCTGCTAACGCACCATA	AAGTCTGTCCGTCGCTGAA
Rhizoctonia solani mitovirus 45	TAGCCATAAGCCACCATA	TCCGAAAGGGAGAAGCAC
Rhizoctonia solani mitovirus 46	AAATTACCCGAAGATGGT	AAGATGAATAGGCTCCCA
Rhizoctonia solani mitovirus 47	GTCTCACCTGTGGCTTTT	GTGTCTGATGCGGTATGT
Rhizoctonia solani mitovirus 48	TGAGACGCAGACGCAGTA	CGGCAGAATCATTAGTAGCT
Rhizoctonia solani mitovirus 49	CCTGTCTCAAAGGCGTAG	TAACGGTTGGAAGTAGGA
Rhizoctonia solani mitovirus 50	TGTCACGATTCTTACCCT	CTCCATCCCATTATTACC
Rhizoctonia solani mitovirus 51	ACAAGGGATGCCCAAACT	TAAAGCGTCCTGCTGTCG
Rhizoctonia solani mitovirus 52	GATCAACGAAGCTCACCC	CCAGGCGTAGAAGCAGAA
Rhizoctonia solani mitovirus 53	TTGGGATGGAACAAACGA	GTATTGAATTAGCCGCACAG
Rhizoctonia solani mitovirus 54	AGCACGGAAGGAGATGTT	GTTTCAATGGGTCGGTAG
Rhizoctonia solani mitovirus 55	TTTGTCATCCTACCCTCT	CTATAACGCCTGGTCCTC

Table S1. (Continued from preceding page).

Name of mycovirus	Sequence of forward primer (5´-3´)	Sequence of reward primer (5´-3´)
Rhizoctonia solani mitovirus 56	AACGGAAGTGTACGATGAG	CTGCCAGGCTGAAATAAC
Rhizoctonia solani mitovirus 57	TTTATCCGCAGTCGTTGT	GTCAGTGGGTTCCTATTT
Rhizoctonia solani mitovirus 58	CCAACAATCCTACCTCCT	CTACCGACAACTTTCCAC
Rhizoctonia solani mitovirus 59	CCACCATCTAATCGTGCA	TCTTCAACCGTGACAGGC
Rhizoctonia solani mitovirus 60	ATTATGTATCTGCCACCC	TGCCCTGATTCTTACTCT
Rhizoctonia solani mitovirus 61	GCTACTGCGGCTTATTCT	CGGCGACAAACTCTGTTA
Rhizoctonia solani mitovirus 62	CCCTGCTGCTTCTTCTTT	ATCCTGGTTCTATTCCGTTTA
Rhizoctonia solani mitovirus 63	CTCCACTGGCTCCATCTA	TACTTACTGGGACCTCAC
Rhizoctonia solani mitovirus 64	AAGAATGCGTGAGTGCTG	GAAGATGAGAATCCCGAAGA
Rhizoctonia solani mitovirus 65	AACCTCTGGACGCTCTTG	GTTCTGATTTGGCTTGGC
Rhizoctonia solani mitovirus 66	GCAACCTATGGGTGCTCT	GCATTACCGTTCTTAGCG
Rhizoctonia solani mitovirus 67	CTAACGACGAAGTAGCAG	AAACACTTTGAGGCAGAA
Rhizoctonia solani mitovirus 68	CAATCTATCCTTGCTCCT	CACTTGTGCTACCGAAAT
Rhizoctonia solani mitovirus 69	ATCAGTTGCGTTGCGATA	AAGAGCATTTGCGTCCAG
Rhizoctonia solani mitovirus 70	GAGTTCCCTGGCACCTTG	TACCCGCAGCCTCATCCT
Rhizoctonia solani mitovirus 71	CTAAAGACGATAACGGAAA	CCTGATAGGATAGCCACA
Rhizoctonia solani mitovirus 72	GTTTACAGCAAACCCAAA	ATCCCTAACTCCGTCATT
Rhizoctonia solani mitovirus 73	CCTATGGGAGCGTTTAGC	ACCGTTTACCTTCCGTCTT
Rhizoctonia solani mitovirus 74	ATGGAGGAAGATGTGGAA	TACCGAGAAGGAGGGACT
Rhizoctonia solani mitovirus 75	CAATTAGGAACCGAAAGC	AGAGCGGAATGAAAGATC
Rhizoctonia solani mitovirus 76	CGGGTTTCAAAGGCTGGT	ATCTTCTACTGCTACGGGTGT
Rhizoctonia solani mitovirus 77	AGAGCGTAAACTTTGTGG	ACTAACTTGGCTTGGAGA
Rhizoctonia solani mitovirus 78	GCTATCGCTAAGTGGAGTA	TTTGGGTCAGTTAAGTTATG
Rhizoctonia solani mitovirus 80	AATTCCGTAATGCGAGAA	TAATAAATGTCACCGCTTGT

Table S1. (Continued from preceding page).

Name of mycovirus	Sequence of forward primer (5´-3´)	Sequence of reward primer (5´-3´)
Rhizoctonia solani mitovirus 81	CATCGGGCTAATAAGACA	GCACTATCCCAGACCAAA
Rhizoctonia solani mitovirus 82	CGGAGGTAGAGTGGCTAA	TCTGCGGTATGCTCAAAT
Rhizoctonia solani mitovirus 83	GTACTCGGAAGCGTCATC	TCTCGCACAAGTGGTCTG
Rhizoctonia solani mitovirus 84	GAGCAATGCCAAACGATG	AGCACGGAAGGAGATGTA
Rhizoctonia solani mitovirus 85	CTCGGAACGGTCAGAGTG	CGAAGTAACCAGGGAAGT
Rhizoctonia solani mitovirus 86	ACCCACCACTGGATTGTTC	GCTGTCATTACACCGCTTAG
Rhizoctonia solani mitovirus 87	AAGAAGAAGCAGCAGGGA	GCCATAGCCGTTACAAGC
Rhizoctonia solani mitovirus 88	ATAAATGGCCCAACAAAG	TCAACAATCCTCGCTCTG
Rhizoctonia solani mitovirus 89	CCCTTATGCTCGGTTTAG	GGACGGGATAGGACAACA
Rhizoctonia solani mitovirus 90	TAGATGGTTAGGCAGTCA	AGCATAAAGCCCAGTGTA
Rhizoctonia solani mitovirus 91	GAGTATTAGGAGGGTTGC	TTATTCAGAGCCCGTTTA
Rhizoctonia solani mitovirus 92	GGAACGAACCGAGGAGTT	CAGCGAAAGCGAGATACAC
Rhizoctonia solani mitovirus 93	GCTTCGCCGTCTAACAAG	CCATACATCGCTCCACAG
Rhizoctonia solani mitovirus 94	GGGTCCAACGCATACTCT	TCCTCCATCATCCCAAAC
Rhizoctonia solani mitovirus 95	AGAGCGAAAGTTCCAAAT	AGCCAACATAGACCAAAG
Rhizoctonia solani mitovirus 96	TAAGTGCCGCTACTGATA	TTCTGAAGGCTGATGTAA
Rhizoctonia solani mitovirus 98	CAATCTATTCAATCTCCCT	TCTTCGGTAATGTCTGTG
Rhizoctonia solani mitovirus 99	CATTTCGTTCATCCAGAC	AATAGCGACAACCCTTAC
Rhizoctonia solani mitovirus 100	TGAAGGTAGTCGGATTGG	GGGGATGCTAAGGAGATA
Rhizoctonia solani mitovirus 101	AAGAAGCGGCAGGTAAAG	AAAAGCGAGACCACGATG
Rhizoctonia solani mitovirus 102	CTCCCTTGTGCCTGAGTA	GACGGTGTATCCCTATGC
Rhizoctonia solani mitovirus 103	CCTATTCTCGCTCATTTA	GTTCTACCGTTATCCTCTG
Rhizoctonia solani mitovirus 104	TTAGTGGACGAGGCGATA	CAAGAGGACTGGAGGGAC
Rhizoctonia solani mitovirus 105	CGTCTCGCATAGACTCAAA	CCACGTTACGAATCCCTC

Table S1. (Continued from preceding page).

Name of mycovirus	Sequence of forward primer (5′-3′)	Sequence of reward primer (5′-3′)
Rhizoctonia solani narnavirus 1	TGGTTTCTCGGCTGATGT	GACGCTGATACTCCTCCC
Rhizoctonia solani narnavirus 2	ACTTTCGTTAGTGGGTCTC	GTCCAGTACAAGCGGTATG
Rhizoctonia solani narnavirus 3	GCCTTCACTCCGTAGATT	CCACTTACCTCCCTGCTC
Rhizoctonia solani narnavirus 4	ATGCCACATTTAAGCATCCACT	CCGTCACGAAAGCCAGGTC
Rhizoctonia solani narnavirus 5	GTGGATGCCAACGCAATT	ACGCAGTCGGGAAGAACG
Rhizoctonia solani narnavirus 6	GCTGAGCAAGGGAATAAG	TCCAGTAGGCAATGAGGT
Rhizoctonia solani narnavirus 7	TTATTCGGCGGATTTGTC	GTGAAGCACCTCCCAGTT
Rhizoctonia solani narnavirus 8	GGATAGGCAGAGGGTCAGTA	TTGTTGGGATAGTAGGTGTCA
Rhizoctonia solani narnavirus 9	CTTCGTCTCCACCTCACC	GTCCTGGGCTCATACTCAA
Rhizoctonia solani narnavirus 10	GTCAAGGAGCCTGGTAAA	GTCTCGCCGTATTCTGGT
Rhizoctonia solani narnavirus 11	CACGTTGTGCAGGTATGA	CACTTATTGCCTTGTTCG
Rhizoctonia solani narnavirus 12	GGACCTTATTGGGCTATCT	TAACGGTTTGCTCTTCTTT
Rhizoctonia solani narnavirus 13	TGAAACACGTACCCAAGC	GGTCCAACTCAATGCCTAA
Rhizoctonia solani narnavirus 14	CGACTTACGACCTTACCC	CGACAAATCTGCCGAATA
Rhizoctonia solani narnavirus 15	TGCTTTACAGTCCACGATT	CATTATGCGATAGCCAGTC
Rhizoctonia solani narnavirus 16	CACGGATATGACCCTACCTC	ACGATTAGACCGACTGGC
Rhizoctonia solani narnavirus 17	TCTTCTCGGTCAGGTTGC	ATCCTATCGGTCGCTTTC
Rhizoctonia solani narnavirus 18	GCTAACGACCCGACTCAA	CGTAATCGCCCTCACAGT
Rhizoctonia solani narnavirus 19	GCAAAGAAGGAGGTCGTAAT	ACCGTGAGTGGGAGAAAT
Rhizoctonia solani ourmia-like virus 7	CGGAGGTAGAGTGGCTAA	TCTGCGGTATGCTCAAAT
Rhizoctonia solani ourmia-like virus 8	CCCGTCCACTCATTCTCC	TTACGATCCGTGACTTACCC
Rhizoctonia solani partitivirus 2 strain beet (CP)	GAGTGAGCGGAGGAAAGG	TCAACAGCATCGCAAACG
Rhizoctonia solani partitivirus 2 strain beet (RdRp)	GCCATCTGAACGCTATTA	GAAAGCACGCAACCCTAC
Rhizoctonia solani partitivirus 15	TCATCACTTGACGGGTAG	ATCTCCGATACTGGCACA

Table S1. (Continued from preceding page).

Name of mycovirus	Sequence of forward primer (5´-3´)	Sequence of reward primer (5´-3´)
Rhizoctonia solani partitivirus 16	TCCATTGCGTGTCCAGTT	CGAATGCGAAATCTCCTC
Rhizoctonia solani partitivirus 17	AGGCGGTGACGGAATACA	ACACTCTTCGTGCCCAAC
Rhizoctonia solani partitivirus 18	TCCGCAAGTAAATGTGAC	TTTCATCAATGGGTGTCG
Rhizoctonia solani partitivirus 19	AGTCTTCACTTGTGCGGTAA	CTCTGGGAATGGGTTTGC
Rhizoctonia solani partitivirus 20	CTTTCGCCATCCTCCTCG	TTCAAAGCCTGACGCTCC
Rhizoctonia solani partitivirus 21	GGGGTCCGTATCTGTGAA	AAGGAAATAGCACGAGGC
Rhizoctonia solani negative-stranded virus 7	AAGCGTTTATTGTCTTTGTC	ATAGCCTTCCTGTGGTCC
Rhizoctonia solani Khurdun virus	GTCAGCCCAGGACATCAA	GGCAAGTCCGTGAACAAA
Rhizoctonia solani narna-like virus 1	CACGGTTGCTCACGACTA	GAAGCGAATCCCAATCCA
Rhizoctonia solani narna-like virus 2	TGGCGAAGACCAGAGGAGA	TGGAACGGAGCCCAATAC
