# Supplementary material for: Anastomosis Groups and Mycovirome of Rhizoctonia Isolates Causing Sugar Beet Root and Crown Rot and Their Sensitivity to Flutolanil, Thifluzamide, and Pencycuron
Source: J Fungi (Basel). 2023 May 9;9(5):545. doi: 10.3390/jof9050545 (PMC10219533; doi:10.3390/jof9050545)
Supplement: Supplementary file 1 [file jof-09-00545-s001.zip › Table S2.pdf]

Table S2. *Rhizoctonia* isolates recovered from sugar beet roots with the symptoms of root and crown rot in China from 2009 to 2016.

Isolate code	Sampling site [z]	Year	AG [y]	GenBank accession number
R1	Suihua city, Heilongjiang province	2009	A	KR259886
R2	Suihua city, Heilongjiang province	2009	A	NM729020
R3	Suihua city, Heilongjiang province	2009	A	NM729021
R4	Heihe city, Heilongjiang province	2009	4HGI	KR259914
R5	Heihe city, Heilongjiang province	2009	2-2IIIB	NM729023
R6	Heihe city, Heilongjiang province	2009	2-2IIIB	KR259889
R7	Qiqihar city, Heilongjiang province	2009	4HGII	KR259934
R8	Suihua city, Heilongjiang province	2009	2-2IIIB	NM729024
R9	Ulanqab city, Inner Mongolia autonomous region	2009	2-2IIIB	KR259890
R10	Ulanqab city, Inner Mongolia autonomous region	2009	2-2IIIB	NM729025
R11	Ulanqab city, Inner Mongolia autonomous region	2009	2-2IIIB	NM729026
R12	Ulanqab city, Inner Mongolia autonomous region	2009	2-2IIIB	NM729027
R13	Ulanqab city, Inner Mongolia autonomous region	2009	2-2IIIB	KR259891
R15	Ulanqab city, Inner Mongolia autonomous region	2009	2-2IIIB	NM729028
R16	Ulanqab city, Inner Mongolia autonomous region	2009	2-2IIIB	NM729029
R17	Chifeng city, Inner Mongolia autonomous region	2009	4HGI	KR259916
R18	Chifeng city, Inner Mongolia autonomous region	2009	4HGI	NM729118
R19	Chifeng city, Inner Mongolia autonomous region	2009	2-2IIIB	KR259892
R20	Chifeng city, Inner Mongolia autonomous region	2009	4HGI	NM729119
R21	Ulanqab city, Inner Mongolia autonomous region	2009	2-2IIIB	NM729031
R23	Qiqihar city, Heilongjiang province	2009	4HGI	NM729032
R24	Qiqihar city, Heilongjiang province	2010	4HGI	NM729120
R25	Qiqihar city, Heilongjiang province	2010	4HGI	NM729121
R26	Mudanjiang city, Heilongjiang province	2010	4HGI	KR259918
R28	Ulanqab city, Inner Mongolia autonomous region	2010	2-2IIIB	KR259893
R29	Ulanqab city, Inner Mongolia autonomous region	2010	2-2IIIB	NM729033
R30	Ulanqab city, Inner Mongolia autonomous region	2010	2-2IIIB	NM729034
R32	Datong city, Shanxi province	2010	2-2IIIB	KR259894
R33	Datong city, Shanxi province	2010	2-2IIIB	NM729035
R35	Haidian district, Beijing municipality	2010	2-2IV	KR259911
R36	Changji city, Xinjiang Uygur autonomous region	2010	2-2IIIB	KR259895
R37	Bayingol city, Xinjiang Uygur autonomous region	2010	4HGI	NM729122
R38	Suihua city, Heilongjiang province	2010	2-2IIIB	KR259896
R39	Qiqihar city, Heilongjiang province	2010	4HGII	KR259935
R40	Qiqihar city, Heilongjiang province	2010	A	NM729022
R41	Baotou city, Inner Mongolia autonomous region	2010	2-2IIIB	KR259897
R42	Chifeng city, Inner Mongolia autonomous region	2010	2-2IIIB	NM729036
RR2	Suihua city, Heilongjiang province	2011	A	KR259888
RR5	Zhangye city, Gansu province	2011	4HGII	KR259939
RR6	Zhangye city, Gansu province	2011	4HGI	KR259924

Table S2. (Continued from preceding page).

Isolate code	Sampling site [z]	Year	AG [y]	GenBank accession number
RR7	Suihua city, Heilongjiang province	2011	4HGII	KR259940
RR8	Qiqihar city, Heilongjiang province	2011	4HGI	KR259925
RR11	Ulanqab city, Inner Mongolia autonomous region	2011	2-2IIIB	KR259903
RR13	Siping city, Jilin province	2011	2-2IIIB	KR259904
RR16	Ulanqab city, Inner Mongolia autonomous region	2011	2-2IIIB	NM729109
RR17	Ulanqab city, Inner Mongolia autonomous region	2011	2-2IIIB	KR259906
RR18	Ili city, Xinjiang Uygur autonomous region	2011	4HGI	NM729176
RR20	Ulanqab city, Inner Mongolia autonomous region	2011	4HGII	KR259941
RR22	Ulanqab city, Inner Mongolia autonomous region	2011	4HGII	NM729210
RR23	Ulanqab city, Inner Mongolia autonomous region	2011	4HGII	KR259942
RR25	Ulanqab city, Inner Mongolia autonomous region	2011	2-2IIIB	KR259908
RR26	Ulanqab city, Inner Mongolia autonomous region	2011	4HGII	NM729211
RR27	Ulanqab city, Inner Mongolia autonomous region	2011	4HGII	KR259943
R1(12)	Qiqihar city, Heilongjiang province	2012	2-2IV	KR259910
R2(12)	Qiqihar city, Heilongjiang province	2012	3 PT	KR259912
R3(12)	Urumqi city, Xinjiang Uygur autonomous region	2012	4HGI	KR259913
R4(12)	Qiqihar city, Heilongjiang province	2012	4HGI	NM729116
R5(12)	Urumqi city, Xinjiang Uygur autonomous region	2012	4HGI	NM729117
R6(12)	Harbin city, Heilongjiang province	2012	4HGI	KR259915
RHB-1	Zhangjiakou city, Hebei province	2013	4HGII	KR259936
RHB-2	Zhangjiakou city, Hebei province	2013	4HGII	NM729205
RHB-3	Zhangjiakou city, Hebei province	2013	4HGII	NM729206
RHB-4	Zhangjiakou city, Hebei province	2013	4HGII	KR259937
RHL1	Qiqihar city, Heilongjiang province	2013	4HGI	KR259919
RHL2	Qiqihar city, Heilongjiang province	2013	4HGI	NM729123
RHL3	Qiqihar city, Heilongjiang province	2013	4HGI	NM729124
RHL4	Qiqihar city, Heilongjiang province	2013	4HGI	NM729125
RHL5	Qiqihar city, Heilongjiang province	2013	4HGI	NM729126
RHL6	Qiqihar city, Heilongjiang province	2013	4HGI	KR259920
RNM-1	Ulanqab city, Inner Mongolia autonomous region	2013	4HGI	NM729175
RNM-2	Ulanqab city, Inner Mongolia autonomous region	2013	4-HGII	KR259938
RNM-3	Ulanqab city, Inner Mongolia autonomous region	2013	4HGI	KR259923
RNM-4	Ulanqab city, Inner Mongolia autonomous region	2013	2-2IIIB	KR259900
RNM-5	Ulanqab city, Inner Mongolia autonomous region	2013	2-2IIIB	NM729106
RNM-7	Ulanqab city, Inner Mongolia autonomous region	2013	2-2IIIB	NM729107
RNM-8	Ulanqab city, Inner Mongolia autonomous region	2013	2-2IIIB	NM729108
RNM-9	Ulanqab city, Inner Mongolia autonomous region	2013	2-2IIIB	KR259901
RSX-1	Datong city, Shanxi province	2013	4HGI	KR259926
RSX-2	Datong city, Shanxi province	2013	4HGI	NM729177
RSX-3	Datong city, Shanxi province	2013	4HGI	KR259927

Table S2. (Continued from preceding page).

Isolate code	Sampling site [z]	Year	AG [y]	GenBank accession number
RSX-5	Datong city, Shanxi province	2013	4HGI	NM729179
RSX-4	Datong city, Shanxi province	2013	4HGI	NM729178
RSX-6	Datong city, Shanxi province	2013	4HGI	NM729180
RSX-7	Datong city, Shanxi province	2013	4HGI	KR259928
RX-1	Changji city, Xinjiang Uygur autonomous region	2013	4HGI	KR259929
RX-2	Changji city, Xinjiang Uygur autonomous region	2013	4HGII	KR259944
RX-3	Changji city, Xinjiang Uygur autonomous region	2013	4HGI	NM729181
RX-4	Changji city, Xinjiang Uygur autonomous region	2013	4HGI	KR259930
RX-5	Changji city, Xinjiang Uygur autonomous region	2013	4HGI	NM729182
RX6	Changji city, Xinjiang Uygur autonomous region	2013	4HGI	NM729183
RX7	Changji city, Xinjiang Uygur autonomous region	2013	4HGI	NM729184
RX8	Changji city, Xinjiang Uygur autonomous region	2013	4HGI	NM729185
RX9	Changji city, Xinjiang Uygur autonomous region	2013	4HGI	NM729186
RX10	Changji city, Xinjiang Uygur autonomous region	2013	4HGI	KR259931
RX11	Shihezi city, Xinjiang Uygur autonomous region	2013	4HGI	NM729187
RX12	Shihezi city, Xinjiang Uygur autonomous region	2013	4HGI	NM729188
RX13	Shihezi city, Xinjiang Uygur autonomous region	2013	4HGI	NM729189
RX14	Shihezi city, Xinjiang Uygur autonomous region	2013	4HGI	NM729190
RX15	Shihezi city, Xinjiang Uygur autonomous region	2013	4HGI	NM729191
RX16	Shihezi city, Xinjiang Uygur autonomous region	2013	4HGI	NM729192
RX17	Shihezi city, Xinjiang Uygur autonomous region	2013	4HGI	KR259932
RN5	Ulanqab city, Inner Mongolia autonomous region	2014	4HGI	KR259922
RN6	Ulanqab city, Inner Mongolia autonomous region	2014	2-2IIIB	NM729037
RN10	Chifeng city, Inner Mongolia autonomous region	2014	2-2IIIB	KR259899
RXJ3	Shihezi city, Xinjiang Uygur autonomous region	2014	4HGI	NM729204
RHL9	Qiqihar city, Heilongjiang province	2015	4HGI	NM729127
RHL10	Qiqihar city, Heilongjiang province	2015	4HGI	NM729128
RHL11	Qiqihar city, Heilongjiang province	2015	4HGI	NM729129
RHL17	Qiqihar city, Heilongjiang province	2015	4HGI	NM729130
RN11	Ulanqab city, Inner Mongolia autonomous region	2015	4HGIII	NM729212
RN15	Ulanqab city, Inner Mongolia autonomous region	2015	2-2IIIB	NM729038
RN17	Ulanqab city, Inner Mongolia autonomous region	2015	K	NM729213
RN19	Ulanqab city, Inner Mongolia autonomous region	2015	K	NM729214
RN20	Ulanqab city, Inner Mongolia autonomous region	2015	4HGI	NM729131
RN21	Ulanqab city, Inner Mongolia autonomous region	2015	4HGI	NM729132
RN22	Ulanqab city, Inner Mongolia autonomous region	2015	4HGI	NM729133
RN23	Ulanqab city, Inner Mongolia autonomous region	2015	4HGI	NM729134
RN25	Ulanqab city, Inner Mongolia autonomous region	2015	4HGI	NM729135
RN28	Hinggan league, Inner Mongolia autonomous region	2015	2-2IIIB	NM729039
RN29	Hinggan league, Inner Mongolia autonomous region	2015	2-2IIIB	NM729040

Table S2. (Continued from preceding page).

Isolate code	Sampling site [z]	Year	AG [y]	GenBank accession number
RN31	Hinggan league, Inner Mongolia autonomous region	2015	2-2IIIB	NM729041
RN39	Hinggan league, Inner Mongolia autonomous region	2015	2-2IIIB	NM729042
RN43	Hinggan league, Inner Mongolia autonomous region	2015	2-2IIIB	NM729043
RN44	Hinggan league, Inner Mongolia autonomous region	2015	2-2IIIB	NM729044
RN49	Hinggan league, Inner Mongolia autonomous region	2015	2-2IIIB	NM729045
RN50	Hinggan league, Inner Mongolia autonomous region	2015	2-2IIIB	NM729046
RN53	Hinggan league, Inner Mongolia autonomous region	2015	2-2IIIB	NM729047
RN56	Hinggan league, Inner Mongolia autonomous region	2015	2-2IIIB	NM729048
RN57	Hinggan league, Inner Mongolia autonomous region	2015	2-2IIIB	NM729049
RN60	Hinggan league, Inner Mongolia autonomous region	2015	2-2IIIB	NM729050
RN61	Hinggan league, Inner Mongolia autonomous region	2015	2-2IIIB	NM729051
RN62	Hinggan league, Inner Mongolia autonomous region	2015	2-2IIIB	NM729052
RN63	Hinggan league, Inner Mongolia autonomous region	2015	2-2IIIB	NM729053
RN65	Hinggan league, Inner Mongolia autonomous region	2015	2-2IIIB	NM729054
RN66	Hinggan league, Inner Mongolia autonomous region	2015	2-2IIIB	NM729055
RN67	Hinggan league, Inner Mongolia autonomous region	2015	2-2IIIB	NM729056
RN69	Hinggan league, Inner Mongolia autonomous region	2015	2-2IIIB	NM729057
RN72	Hinggan league, Inner Mongolia autonomous region	2015	2-2IIIB	NM729058
RN74	Hinggan league, Inner Mongolia autonomous region	2015	2-2IIIB	NM729059
RN76	Hinggan league, Inner Mongolia autonomous region	2015	2-2IIIB	NM729060
RN78	Chifeng city, Inner Mongolia autonomous region	2015	4HGI	NM729136
RN79	Chifeng city, Inner Mongolia autonomous region	2015	4HGI	NM729137
RN80	Chifeng city, Inner Mongolia autonomous region	2015	4HGI	NM729138
RN81	Chifeng city, Inner Mongolia autonomous region	2015	4HGI	NM729139
RN82	Chifeng city, Inner Mongolia autonomous region	2015	4HGI	NM729140
RN83	Chifeng city, Inner Mongolia autonomous region	2015	4HGI	NM729141
RN84	Chifeng city, Inner Mongolia autonomous region	2015	4HGI	NM729142
RN85	Chifeng city, Inner Mongolia autonomous region	2015	4HGI	NM729143
RN86	Chifeng city, Inner Mongolia autonomous region	2015	4HGI	NM729144
RN87	Chifeng city, Inner Mongolia autonomous region	2015	4HGI	NM729145
RN88	Chifeng city, Inner Mongolia autonomous region	2015	4HGI	NM729146
RN90	Chifeng city, Inner Mongolia autonomous region	2015	4HGI	NM729147
RN91	Chifeng city, Inner Mongolia autonomous region	2015	2-2IIIB	NM729061
RN92	Chifeng city, Inner Mongolia autonomous region	2015	2-2IIIB	NM729062
RN94	Chifeng city, Inner Mongolia autonomous region	2015	2-2IIIB	NM729063
RN95	Chifeng city, Inner Mongolia autonomous region	2015	2-2IIIB	NM729064
RN97	Chifeng city, Inner Mongolia autonomous region	2015	2-2IIIB	NM729065
RN98	Chifeng city, Inner Mongolia autonomous region	2015	2-2IIIB	NM729066
RN99	Chifeng city, Inner Mongolia autonomous region	2015	2-2IIIB	NM729067
RN100	Chifeng city, Inner Mongolia autonomous region	2015	2-2IIIB	NM729068

Table S2. (Continued from preceding page).

Isolate code	Sampling site [z]	Year	AG [y]	GenBank accession number
RN102	Chifeng city, Inner Mongolia autonomous region	2015	2-2IIIB	NM729069
RN104	Chifeng city, Inner Mongolia autonomous region	2015	2-2IIIB	NM729070
RN105	Hohhot city, Inner Mongolia autonomous region	2015	4HGI	NM729148
RX18	Urumqi city, Xinjiang Uygur autonomous region	2015	4HGI	NM729193
RX19	Urumqi city, Xinjiang Uygur autonomous region	2015	4HGI	NM729194
RX20	Urumqi city, Xinjiang Uygur autonomous region	2015	4HGI	NM729195
RX21	Urumqi city, Xinjiang Uygur autonomous region	2015	4HGI	NM729196
RX22	Urumqi city, Xinjiang Uygur autonomous region	2015	4HGI	NM729197
RX23	Urumqi city, Xinjiang Uygur autonomous region	2015	4HGI	NM729198
RX24	Urumqi city, Xinjiang Uygur autonomous region	2015	4HGI	NM729199
RX25	Urumqi city, Xinjiang Uygur autonomous region	2015	4HGI	NM729200
RX26	Urumqi city, Xinjiang Uygur autonomous region	2015	4HGI	NM729201
RX28	Ili city, Xinjiang Uygur autonomous region	2015	2-2IIIB	NM729110
RX29	Ili city, Xinjiang Uygur autonomous region	2015	2-2IIIB	NM729111
RX31	Urumqi city, Xinjiang Uygur autonomous region	2015	2-2IIIB	NM729112
RX39	Changji city, Xinjiang Uygur autonomous region	2015	4HGI	NM729202
RX44	Changji city, Xinjiang Uygur autonomous region	2015	4HGI	NM729203
RN106	Ulanqab city, Inner Mongolia autonomous region	2016	4HGI	NM729149
RN107	Ulanqab city, Inner Mongolia autonomous region	2016	4HGI	NM729150
RN108	Ulanqab city, Inner Mongolia autonomous region	2016	4HGI	NM729151
RN109	Ulanqab city, Inner Mongolia autonomous region	2016	4HGI	NM729152
RN110	Ulanqab city, Inner Mongolia autonomous region	2016	4HGI	NM729153
RN111	Ulanqab city, Inner Mongolia autonomous region	2016	4HGI	NM729154
RN112	Ulanqab city, Inner Mongolia autonomous region	2016	4HGI	NM729155
RN113	Ulanqab city, Inner Mongolia autonomous region	2016	4HGI	NM729156
RN114	Ulanqab city, Inner Mongolia autonomous region	2016	2-2IIIB	NM729071
RN115	Ulanqab city, Inner Mongolia autonomous region	2016	2-2IIIB	NM729072
RN116	Ulanqab city, Inner Mongolia autonomous region	2016	2-2IIIB	NM729073
RN117	Ulanqab city, Inner Mongolia autonomous region	2016	2-2IIIB	NM729074
RN118	Ulanqab city, Inner Mongolia autonomous region	2016	2-2IIIB	NM729075
RN119	Ulanqab city, Inner Mongolia autonomous region	2016	2-2IIIB	NM729076
RN120	Ulanqab city, Inner Mongolia autonomous region	2016	2-2IIIB	NM729077
RN121	Ulanqab city, Inner Mongolia autonomous region	2016	2-2IIIB	NM729078
RN122	Ulanqab city, Inner Mongolia autonomous region	2016	2-2IIIB	NM729079
RN123	Chifeng city, Inner Mongolia autonomous region	2016	4HGI	NM729157
RN124	Chifeng city, Inner Mongolia autonomous region	2016	4HGI	NM729158
RN125	Chifeng city, Inner Mongolia autonomous region	2016	4HGI	NM729159
RN126	Chifeng city, Inner Mongolia autonomous region	2016	2-2IIIB	NM729080
RN127	Chifeng city, Inner Mongolia autonomous region	2016	4HGI	NM729160
RN128	Chifeng city, Inner Mongolia autonomous region	2016	4HGI	NM729161

Table S2. (Continued from preceding page).

Isolate code	Sampling site [z]	Year	AG [y]	GenBank accession number
RN129	Chifeng city, Inner Mongolia autonomous region	2016	2-2IIIB	NM729081
RN130	Chifeng city, Inner Mongolia autonomous region	2016	2-2IIIB	NM729082
RN131	Chifeng city, Inner Mongolia autonomous region	2016	2-2IIIB	NM729083
RN132	Chifeng city, Inner Mongolia autonomous region	2016	2-2IIIB	NM729084
RN133	Chifeng city, Inner Mongolia autonomous region	2016	4HGI	NM729162
RN134	Chifeng city, Inner Mongolia autonomous region	2016	4HGI	NM729163
RN135	Chifeng city, Inner Mongolia autonomous region	2016	4HGI	NM729164
RN136	Chifeng city, Inner Mongolia autonomous region	2016	4HGI	NM729165
RN137	Chifeng city, Inner Mongolia autonomous region	2016	4HGI	NM729166
RN138	Chifeng city, Inner Mongolia autonomous region	2016	4HGI	NM729167
RN139	Chifeng city, Inner Mongolia autonomous region	2016	4HGI	NM729168
RN140	Chifeng city, Inner Mongolia autonomous region	2016	4HGI	NM729169
RN141	Chifeng city, Inner Mongolia autonomous region	2016	4HGI	NM729170
RN142	Chifeng city, Inner Mongolia autonomous region	2016	4HGI	NM729171
RN143	Chifeng city, Inner Mongolia autonomous region	2016	4HGI	NM729172
RN144	Chifeng city, Inner Mongolia autonomous region	2016	4HGI	NM729173
RN145	Chifeng city, Inner Mongolia autonomous region	2016	4HGI	NM729174
RN146	Ulanqab city, Inner Mongolia autonomous region	2016	2-2IIIB	NM729085
RN147	Ulanqab city, Inner Mongolia autonomous region	2016	2-2IIIB	NM729086
RN148	Ulanqab city, Inner Mongolia autonomous region	2016	2-2IIIB	NM729087
RN149	Ulanqab city, Inner Mongolia autonomous region	2016	2-2IIIB	NM729088
RN150	Ulanqab city, Inner Mongolia autonomous region	2016	2-2IIIB	NM729089
RN151	Ulanqab city, Inner Mongolia autonomous region	2016	2-2IIIB	NM729090
RN152	Ulanqab city, Inner Mongolia autonomous region	2016	2-2IIIB	NM729091
RN153	Ulanqab city, Inner Mongolia autonomous region	2016	2-2IIIB	NM729092
RN154	Ulanqab city, Inner Mongolia autonomous region	2016	2-2IIIB	NM729093
RN155	Ulanqab city, Inner Mongolia autonomous region	2016	2-2IIIB	NM729094
RN156	Ulanqab city, Inner Mongolia autonomous region	2016	2-2IIIB	NM729095
RN157	Ulanqab city, Inner Mongolia autonomous region	2016	2-2IIIB	NM729096
RN158	Ulanqab city, Inner Mongolia autonomous region	2016	2-2IIIB	NM729097
RN159	Ulanqab city, Inner Mongolia autonomous region	2016	2-2IIIB	NM729098
RN160	Ulanqab city, Inner Mongolia autonomous region	2016	2-2IIIB	NM729099
RN161	Ulanqab city, Inner Mongolia autonomous region	2016	2-2IIIB	NM729100
RN162	Ulanqab city, Inner Mongolia autonomous region	2016	2-2IIIB	NM729101
RN163	Chifeng city, Inner Mongolia autonomous region	2016	2-2IIIB	NM729102
RN164	Chifeng city, Inner Mongolia autonomous region	2016	2-2IIIB	NM729103
RN165	Chifeng city, Inner Mongolia autonomous region	2016	2-2IIIB	NM729104
RN166	Chifeng city, Inner Mongolia autonomous region	2016	2-2IIIB	NM729105
RN167	Ulanqab city, Inner Mongolia autonomous region	2016	4HGII	NM729207
RN168	Ulanqab city, Inner Mongolia autonomous region	2016	4HGII	NM729208

Table S2. (Continued from preceding page).

Isolate code	Sampling site [z]	Year	AG [y]	GenBank accession number
RN169	Ulanqab city, Inner Mongolia autonomous region	2016	4HGII	NM729209
RX46	Changji city, Xinjiang Uygur autonomous region	2016	2-2IIIB	NM729113
RX47	Changji city, Xinjiang Uygur autonomous region	2016	2-2IIIB	NM729114
RX48	Changji city, Xinjiang Uygur autonomous region	2016	2-2IIIB	NM729115

Note: [z] Geographic origins where the *Rhizoctonia* isolates were collected in China. [y] Anastomosis groups or subgroups of *Rhizoctonia.*
