# Supplementary material for: Anastomosis Groups and Mycovirome of Rhizoctonia Isolates Causing Sugar Beet Root and Crown Rot and Their Sensitivity to Flutolanil, Thifluzamide, and Pencycuron
Source: J Fungi (Basel). 2023 May 9;9(5):545. doi: 10.3390/jof9050545 (PMC10219533; doi:10.3390/jof9050545)
Supplement: Supplementary file 1 [file jof-09-00545-s001.zip › Table S3.pdf]

Table S3. Assembled sequences of mycoviruses found in *Rhizoctonia* isolates associated with sugar beet root and crown rot and their amino acid (aa) identities to those of viruses described previously.

Contig ID	Name of mycovirus [z]	GenBank accession number	Contig length (nt)	Best match	Identity (%, aa)	Genome type	Number of reads	Family	Number of hosts
Contig 21663	Rhizoctonia solani beny-like virus 2 (RsBLV2)	MZ043902	1982	Wheat stripe mosaic virus	21.44	+ssRNA	53	*Benyviridae*	10
Contig 38678	Rhizoctonia solani beny-like virus 3 (RsBLV3)	MZ043903	1049	Rhizoctonia solani beny-like virus 1	96.40	+ssRNA	9	*Benyviridae*	19
First_Contig 592	Rhizoctonia solani beny-like virus 4 (RsBLV4)	MZ043904	1325	Wheat stripe mosaic virus	32.52	+ssRNA	1242	*Benyviridae*	14
First_Contig 680	Rhizoctonia solani beny-like virus 5 (RsBLV5)	MZ043905	2258	Rice stripe necrosis virus	30.58	+ssRNA	1141	*Benyviridae*	13
First_Contig 796	Rhizoctonia solani beny-like virus 6 (RsBLV6)	MZ043906	7462	Hubei Beny-like virus 1	27.20	+ssRNA	88	*Benyviridae*	5
Contig 11890	Rhizoctonia solani hypovirus 5 (RsHV5)	MZ043907	1185	Sclerotium rolfsii hypovirus 8	44.39	+ssRNA	209	*Hypoviridae*	3
Contig 41165	Rhizoctonia solani hypovirus 6 (RsHV6)	MZ043908	1673	Sclerotinia homoeocarpa hypovirus 1	33.10	+ssRNA	20	*Hypoviridae*	1
First_Contig 2556	Rhizoctonia solani hypovirus 7 (RsHV7)	MZ043909	4484	Sclerotium rolfsii hypovirus 8	34.27	+ssRNA	53	*Hypoviridae*	3
Contig 7027	Rhizoctonia solani hypovirus 8 (RsHV8)	MZ043910	923	Rhizoctonia solani hypovirus 1	55.41	+ssRNA	21	*Hypoviridae*	15
Contig 98	Rhizoctonia solani mitovirus 41 (RsMV41)	MZ043925	2688	Rhizoctonia solani mitovirus 31	77.00	+ssRNA	126	*Mitoviridae*	12
Contig 190	Rhizoctonia solani mitovirus 42 (RsMV42)	MZ043926	4172	Rhizoctonia solani mitovirus 14	57.91	+ssRNA	7017	*Mitoviridae*	17
Contig 195	Rhizoctonia solani mitovirus 43 (RsMV43)	MZ043927	3696	Epicoccum nigrum mitovirus 1	53.20	+ssRNA	1422	*Mitoviridae*	6
Contig 249	Rhizoctonia solani mitovirus 44 (RsMV44)	MZ043928	2659	Neofusicoccum parvum mitovirus 1	88.52	+ssRNA	18	*Mitoviridae*	2
Contig 276	Rhizoctonia solani mitovirus 45 (RsMV45)	MZ043929	1990	Neofusicoccum parvum mitovirus 1	70.05	+ssRNA	317	*Mitoviridae*	9

Contig 330	Rhizoctonia solani mitovirus 46 (RsMV46)	MZ043930	1035	Rhizoctonia cerealis mitovirus	87.50	+ssRNA	25	*Mitoviridae*	3

Table S3. (Continued from preceding page).

Contig ID	Name of mycovirus [z]	GenBank accession number	Contig length (nt)	Best match	Identity (%, aa)	Genome type	Number of reads	Family	Number of hosts
Contig 370	Rhizoctonia solani mitovirus 47 (RsMV47)	MZ043931	3211	Macrophomina phaseolina mitovirus 3	41.43	+ssRNA	1484	*Mitoviridae*	1
Contig 466	Rhizoctonia solani mitovirus 48 (RsMV48)	MZ043932	4397	Rhizoctonia solani mitovirus 21	58.70	+ssRNA	11605	*Mitoviridae*	1
Contig 468	Rhizoctonia solani mitovirus 49 (RsMV49)	MZ043933	4377	Rhizoctonia solani mitovirus 21	76.89	+ssRNA	54076	*Mitoviridae*	1
Contig 508	Rhizoctonia solani mitovirus 50 (RsMV50)	MZ043934	3888	Mitovirus sp.	42.71	+ssRNA	1139	*Mitoviridae*	1
Contig 509	Rhizoctonia solani mitovirus 51 (RsMV51)	MZ043935	3899	Mitovirus sp.	43.61	+ssRNA	46	*Mitoviridae*	3
Contig 538	Rhizoctonia solani mitovirus 52 (RsMV52)	MZ043936	2552	Rhizoctonia solani mitovirus 26	84.54	+ssRNA	13	*Mitoviridae*	2
Contig 539	Rhizoctonia solani mitovirus 53 (RsMV53)	MZ043937	3034	Rhizoctonia solani mitovirus 26	85.06	+ssRNA	67	*Mitoviridae*	1
Contig 604	Rhizoctonia solani mitovirus 54 (RsMV54)	MZ043938	3669	Mitovirus sp.	48.70	+ssRNA	112	*Mitoviridae*	2
Contig 744	Rhizoctonia solani mitovirus 55 (RsMV55)	MZ043939	1616	Macrophomina phaseolina mitovirus 3	39.83	+ssRNA	5226	*Mitoviridae*	2
Contig 801	Rhizoctonia solani mitovirus 56 (RsMV56)	MZ043940	1141	Alternaria alternata mitovirus 1	78.89	+ssRNA	396	*Mitoviridae*	12
Contig 851	Rhizoctonia solani mitovirus 57 (RsMV57)	MZ043941	3173	Rhizoctonia solani mitovirus 8	72.80	+ssRNA	74	*Mitoviridae*	2
Contig 997	Rhizoctonia solani mitovirus 58 (RsMV58)	MZ043942	3398	Rhizoctonia solani mitovirus 34	80.92	+ssRNA	3109	*Mitoviridae*	1
Contig 1038	Rhizoctonia solani mitovirus 59 (RsMV59)	MZ043943	4011	Rhizoctonia solani mitovirus 32	70.90	+ssRNA	11127	*Mitoviridae*	1
Contig 1104	Rhizoctonia solani mitovirus 60 (RsMV60)	MZ043944	3539	Epicoccum nigrum mitovirus 1	75.54	+ssRNA	92	*Mitoviridae*	1
Contig 1212	Rhizoctonia solani mitovirus 61 (RsMV61)	MZ043946	1797	Rhizoctonia solani mitovirus 37	77.25	+ssRNA	3547	*Mitoviridae*	2

Contig 1608	Rhizoctonia solani mitovirus 62 (RsMV62)	MZ043947	1144	Rhizoctonia solani mitovirus 33	58.02	+ssRNA	2912	*Mitoviridae*	6

Table S3. (Continued from preceding page).

Contig ID	Name of mycovirus [z]	GenBank accession number	Contig length (nt)	Best match	Identity (%, aa)	Genome type	Number of reads	Family	Number of hosts
Contig 1830	Rhizoctonia solani mitovirus 63 (RsMV63)	MZ043948	3182	Rhizoctonia solani mitovirus 27	77.09	+ssRNA	534	*Mitoviridae*	1
Contig 2377	Rhizoctonia solani mitovirus 64 (RsMV64)	MZ043949	4296	Rhizoctonia solani mitovirus 1	72.18	+ssRNA	87	*Mitoviridae*	1
Contig 3007	Rhizoctonia solani mitovirus 65 (RsMV65)	MZ043950	3757	Rhizoctonia solani mitovirus 25	62.19	+ssRNA	418	*Mitoviridae*	2
Contig 3241	Rhizoctonia solani mitovirus 66 (RsMV66)	MZ043951	3530	Epicoccum nigrum mitovirus 1	85.00	+ssRNA	2123	*Mitoviridae*	2
Contig 3312	Rhizoctonia solani mitovirus 67 (RsMV67)	MZ043952	2140	Neofusicoccum parvum mitovirus 1	46.21	+ssRNA	1591	*Mitoviridae*	1
Contig 3460	Rhizoctonia solani mitovirus 68 (RsMV68)	MZ043953	1122	Alternaria alternata mitovirus 1	77.35	+ssRNA	1047	*Mitoviridae*	1
Contig 3465	Rhizoctonia solani mitovirus 69 (RsMV69)	MZ043954	2915	Rhizoctonia solani mitovirus 26	82.62	+ssRNA	1033	*Mitoviridae*	6
Contig 4699	Rhizoctonia solani mitovirus 70 (RsMV70)	MZ043955	1910	Rhizoctonia solani mitovirus 37	86.17	+ssRNA	337	*Mitoviridae*	2
Contig 10147	Rhizoctonia solani mitovirus 71 (RsMV71)	MZ043959	2937	Mitovirus sp.	89.66	+ssRNA	453	*Mitoviridae*	1
Contig 32231	Rhizoctonia solani mitovirus 72 (RsMV72)	MZ043966	1338	Ceratobasidium mitovirus A	69.51	+ssRNA	9	*Mitoviridae*	5
First_Contig 14	Rhizoctonia solani mitovirus 73 (RsMV73)	MZ043967	2568	Macrophomina phaseolina mitovirus 3	42.95	+ssRNA	3276	*Mitoviridae*	4
First_Contig 16	Rhizoctonia solani mitovirus 74 (RsMV74)	MZ043968	1891	Rhizoctonia solani mitovirus 21	56.37	+ssRNA	1693	*Mitoviridae*	15
First_Contig 20	Rhizoctonia solani mitovirus 75 (RsMV75)	MZ043969	2621	Macrophomina phaseolina mitovirus 3	42.97	+ssRNA	4886	*Mitoviridae*	9
First_Contig 22	Rhizoctonia solani mitovirus 76 (RsMV76)	MZ043970	3021	Rhizoctonia solani mitovirus 31	76.02	+ssRNA	2396	*Mitoviridae*	17
First_Contig 24	Rhizoctonia solani mitovirus 77 (RsMV77)	MZ043971	1590	Rhizoctonia solani mitovirus 38	83.38	+ssRNA	1498	*Mitoviridae*	9

First_Contig 27	Rhizoctonia solani mitovirus 78 (RsMV78)	MZ043972	3564	Alternaria alternata mitovirus 1	80.87	+ssRNA	13315	*Mitoviridae*	5

Table S3. (Continued from preceding page).

Contig ID	Name of mycovirus [z]	GenBank accession number	Contig length (nt)	Best match	Identity (%, aa)	Genome type	Number of reads	Family	Number of hosts
First_Contig 40	Rhizoctonia solani mitovirus 80 (RsMV80)	MZ043974	2633	Rhizoctonia solani mitovirus 31	71.10	+ssRNA	4424	*Mitoviridae*	8
First_Contig 65	Rhizoctonia solani mitovirus 81 (RsMV81)	MZ043975	1198	Rhizoctonia solani mitovirus 25	54.11	+ssRNA	3978	*Mitoviridae*	12
First_Contig 78	Rhizoctonia solani mitovirus 82 (RsMV82)	MZ043976	2782	Rhizoctonia solani mitovirus 22	51.80	+ssRNA	3020	*Mitoviridae*	2
First_Contig 139	Rhizoctonia solani mitovirus 83 (RsMV83)	MZ043977	3833	Rhizoctonia solani mitovirus 7	70.06	+ssRNA	4744	*Mitoviridae*	12
First_Contig 203	Rhizoctonia solani mitovirus 84 (RsMV84)	MZ043978	3287	Epicoccum nigrum mitovirus 1	47.54	+ssRNA	7275	*Mitoviridae*	3
First_Contig 219	Rhizoctonia solani mitovirus 85 (RsMV85)	MZ043979	2295	Neofusicoccum parvum mitovirus 1	66.33	+ssRNA	1577	*Mitoviridae*	2
First_Contig 230	Rhizoctonia solani mitovirus 86 (RsMV86)	MZ043980	1560	Alternaria alternata mitovirus 1	70.21	+ssRNA	8959	*Mitoviridae*	10
First_Contig 275	Rhizoctonia solani mitovirus 87 (RsMV87)	MZ043981	3324	Rhizoctonia solani mitovirus 25	47.64	+ssRNA	1129	*Mitoviridae*	14
First_Contig 345	Rhizoctonia solani mitovirus 88 (RsMV88)	MZ043982	4347	Rhizoctonia solani mitovirus 33	62.34	+ssRNA	2134	*Mitoviridae*	1
First_Contig 361	Rhizoctonia solani mitovirus 89 (RsMV89)	MZ043983	1510	Epicoccum nigrum mitovirus 1	40.00	+ssRNA	1691	*Mitoviridae*	1
First_Contig 370	Rhizoctonia solani mitovirus 90 (RsMV90)	MZ043984	1909	Rhizoctonia solani mitovirus 37	85.23	+ssRNA	2883	*Mitoviridae*	3
First_Contig 391	Rhizoctonia solani mitovirus 91 (RsMV91)	MZ043985	1155	Rhizoctonia solani mitovirus 31	73.70	+ssRNA	495	*Mitoviridae*	11
First_Contig 618	Rhizoctonia solani mitovirus 92 (RsMV92)	MZ043987	1531	Macrophomina phaseolina mitovirus 3	87.17	+ssRNA	783	*Mitoviridae*	1
First_Contig 723	Rhizoctonia solani mitovirus 93 (RsMV93)	MZ043988	1965	Rhizoctonia solani mitovirus 22	57.20	+ssRNA	176	*Mitoviridae*	1
First_Contig 740	Rhizoctonia solani mitovirus 94 (RsMV94)	MZ043989	2473	Rhizoctonia solani mitovirus 25	45.35	+ssRNA	155	*Mitoviridae*	5

First_Contig 919	Rhizoctonia solani mitovirus 95 (RsMV95)	MZ043991	2763	Rhizoctonia solani mitovirus 21	55.36	+ssRNA	131	*Mitoviridae*	13

Table S3. (Continued from preceding page).

Contig ID	Name of mycovirus [z]	GenBank accession number	Contig length (nt)	Best match	Identity (%, aa)	Genome type	Number of reads	Family	Number of hosts
First_Contig 1173	Rhizoctonia solani mitovirus 96 (RsMV96)	MZ043994	2406	Rhizoctonia solani mitovirus 11	41.36	+ssRNA	403	*Mitoviridae*	1
First_Contig 2276	Rhizoctonia solani mitovirus 98 (RsMV98)	MZ043997	1152	Mitovirus sp.	64.25	+ssRNA	33	*Mitoviridae*	1
First_Contig 4278	Rhizoctonia solani mitovirus 99 (RsMV99)	MZ043998	2336	Rhizoctonia solani mitovirus 23	65.01	+ssRNA	106	*Mitoviridae*	10
First_Contig 6503	Rhizoctonia solani mitovirus 100 (RsMV100)	MZ044000	1246	Rhizoctonia solani mitovirus 38	75.52	+ssRNA	581	*Mitoviridae*	1
Second_Contig 58	Rhizoctonia solani mitovirus 101 (RsMV101)	MZ044001	2603	Macrophomina phaseolina mitovirus 3	86.19	+ssRNA	8	*Mitoviridae*	1
Contig 11664	Rhizoctonia solani mitovirus 102 (RsMV102)	MZ044005	3456	Sclerotinia sclerotiorum mitovirus 11	74.05	+ssRNA	892	*Mitoviridae*	1
Contig 163	Rhizoctonia solani mitovirus 103 (RsMV103)	MZ044006	2886	Rhizoctonia solani mitovirus 2	56.04	+ssRNA	4789	*Mitoviridae*	1
Contig 1141	Rhizoctonia solani mitovirus 104 (RsMV104)	MZ044007	3038	Rhizoctonia solani mitovirus 25	48.38	+ssRNA	10920	*Mitoviridae*	1
Contig 1714	Rhizoctonia solani mitovirus 105 (RsMV105)	MZ044008	3723	Rhizoctonia solani mitovirus 14	52.10	+ssRNA	2204	*Mitoviridae*	1
Contig 1113	Rhizoctonia solani narnavirus 1 (RsNV1)	MZ043945	2273	Fusarium poae narnavirus 1	30.83	+ssRNA	985	*Narnaviridae*	7
Contig 8446	Rhizoctonia solani narnavirus 2 (RsNV2)	MZ043956	1210	Alternaria tenuissima narnavirus 1	53.66	+ssRNA	653	*Narnaviridae*	6
Contig 8527	Rhizoctonia solani narnavirus 3 (RsNV3)	MZ043957	2331	Fusarium poae narnavirus 1	30.71	+ssRNA	1639	*Narnaviridae*	5
Contig 8560	Rhizoctonia solani narnavirus 4 (RsNV4)	MZ043958	2408	Alternaria tenuissima narnavirus 1	45.55	+ssRNA	20	*Narnaviridae*	5
Contig 10382	Rhizoctonia solani narnavirus 5 (RsNV5)	MZ043960	1444	Fusarium poae narnavirus 1	28.94	+ssRNA	317	*Narnaviridae*	1
Contig 11098	Rhizoctonia solani narnavirus 6 (RsNV6)	MZ043961	1038	Fusarium poae narnavirus 1	32.98	+ssRNA	19	Narnaviridae	2

Contig 11100	Rhizoctonia solani narnavirus 7 (RsNV7)	MZ043962	1803	Fusarium poae narnavirus 1	31.72	+ssRNA	92	Narnaviridae	2

Table S3. (Continued from preceding page).

Contig ID	Name of mycovirus [z]	GenBank accession number	Contig length (nt)	Best match	Identity (%, aa)	Genome type	Number of reads	Family	Number of hosts
Contig 15744	Rhizoctonia solani narnavirus 8 (RsNV8)	MZ043963	1645	Fusarium poae narnavirus 1	32.99	+ssRNA	358	*Narnaviridae*	7
Contig 19187	Rhizoctonia solani narnavirus 9 (RsNV9)	MZ043964	2279	Fusarium poae narnavirus 1	28.98	+ssRNA	193	*Narnaviridae*	3
Contig 26242	Rhizoctonia solani narnavirus 10 (RsNV10)	MZ043965	1031	Alternaria tenuissima narnavirus 1	58.50	+ssRNA	12	*Narnaviridae*	1
First_Contig 569	Rhizoctonia solani narnavirus 11 (RsNV11)	MZ043986	1914	Fusarium poae narnavirus 1	29.81	+ssRNA	317	*Narnaviridae*	3
First_Contig 917	Rhizoctonia solani narnavirus 12 (RsNV12)	MZ043990	2296	Fusarium poae narnavirus 1	28.68	+ssRNA	331	*Narnaviridae*	4
First_Contig 1098	Rhizoctonia solani narnavirus 13 (RsNV13)	MZ043992	2235	Fusarium poae narnavirus 1	28.29	+ssRNA	117	*Narnaviridae*	5
First_Contig 1115	Rhizoctonia solani narnavirus 14 (RsNV14)	MZ043993	1279	Fusarium poae narnavirus 1	35.26	+ssRNA	944	*Narnaviridae*	2
First_Contig 1266	Rhizoctonia solani narnavirus 15 (RsNV15)	MZ043996	2226	Fusarium poae narnavirus 1	29.70	+ssRNA	427	*Narnaviridae*	1
First_Contig 5037	Rhizoctonia solani narnavirus 16 (RsNV16)	MZ043999	2406	Fusarium poae narnavirus 1	28.96	+ssRNA	381	*Narnaviridae*	244
Second_Contig 122	Rhizoctonia solani narnavirus 17 (RsNV17)	MZ044002	1521	Fusarium poae narnavirus 1	28.27	+ssRNA	24	*Narnaviridae*	1
Second_Contig 134	Rhizoctonia solani narnavirus 18 (RsNV18)	MZ044003	1824	Fusarium poae narnavirus 1	27.97	+ssRNA	592	*Narnaviridae*	4
Contig 43689	Rhizoctonia solani narnavirus 19 (RsNV19)	MZ044004	1561	Fusarium poae narnavirus 1	28.72	+ssRNA	4	*Narnaviridae*	1
First_Contig 1013	Rhizoctonia solani ourmia-like virus 7 (RsOLV7)	MZ043911	812	Colletotrichum gloeosporioides ourmia-like virus 1	34.68	+ssRNA	16421	*Botourmiaviridae*	2
Contig 3797	Rhizoctonia solani ourmia-like virus 8	MZ043912	619	Rhizoctonia solani ourmia-like	80.00	+ssRNA	37	Botourmiaviridae	9

	(RsOLV8)			virus 1					

Table S3. (Continued from preceding page).

Contig ID	Name of mycovirus [z]	GenBank accession number	Contig length (nt)	Best match	Identity (%, aa)	Genome type	Number of reads	Family	Number of hosts
Contig 2073	Rhizoctonia solani partitivirus 2 strain beet (RsPV2B CP)	MZ043916	1754	Rhizoctonia solani dsRNA virus 2	99.39	dsRNA	1689	Partitiviridae	37
Contig 3529	Rhizoctonia solani partitivirus 2 strain beet (RsPV2B RdRp)	MZ043917	1954	Rhizoctonia solani dsRNA virus 2	99.52	dsRNA	23	*Partitiviridae*	37
Contig 3073	Rhizoctonia solani partitivirus 15 (RsPV15)	MZ043918	1884	Rhizoctonia solani partitivirus 8	67.68	dsRNA	561	*Partitiviridae*	6
Contig 10837	Rhizoctonia solani partitivirus 16 (RsPV16)	MZ043919	1190	Rhizoctonia solani partitivirus 7	54.52	dsRNA	59	*Partitiviridae*	21
Contig 14473	Rhizoctonia solani partitivirus 17 (RsPV17)	MZ043920	1511	Rhizoctonia solani dsRNA virus 3	85.77	dsRNA	179	*Partitiviridae*	13
Contig 14630	Rhizoctonia solani partitivirus 18 (RsPV18)	MZ043921	2011	Fusarium solani partitivirus 2	47.52	dsRNA	28	*Partitiviridae*	10
Contig 14675	Rhizoctonia solani partitivirus 19 (RsPV19)	MZ043922	930	Rhizoctonia solani partitivirus 7	49.32	dsRNA	384	*Partitiviridae*	22
Contig 45845	Rhizoctonia solani partitivirus 20 (RsPV20)	MZ043923	1187	Rosellinia necatrix partitivirus 7	62.10	dsRNA	47	*Partitiviridae*	7
Contig 48291	Rhizoctonia solani partitivirus 21 (RsPV21)	MZ043924	1493	Rhizoctonia solani partitivirus 1	61.49	dsRNA	31	*Partitiviridae*	4
First_Contig 6326	Rhizoctonia solani negative-stranded virus 7 (RsNSV7)	MZ043915	1238	Rhizoctonia solani negative-stranded virus 2	73.54	-ssRNA	58	unclassified	4
Contig 8654	Rhizoctonia solani Khurdun virus (RsKV)	MZ043901	5522	Khurdun virus	27.09	-ssRNA	337	unclassified	4
First_Contig 316	Rhizoctonia solani narna-like virus 1	MZ043913	1747	Wenling narna-like virus 6	24.72	unclassified	4177	unclassified	1

	(RsNLV1)								

Table S3. (Continued from preceding page).

**Contig ID**	**Name of mycovirus** [z]	**GenBank accession number**	**Contig length (nt)**	**Best match**	**Identity (%, aa)**	**Genome type**	**Number of reads**	**Family**	**Number of hosts**
First_Contig 1168	Rhizoctonia solani narna-like virus 2 (RsNLV2)	MZ043914	1655	Wenling narna-like virus 6	26.76	unclassified	150	unclassified	3

Note: [z] Letters followed by Arabic numerals in the parentheses represent the abbreviation of the virus.
