# Supplementary material for: Anastomosis Groups and Mycovirome of Rhizoctonia Isolates Causing Sugar Beet Root and Crown Rot and Their Sensitivity to Flutolanil, Thifluzamide, and Pencycuron
Source: J Fungi (Basel). 2023 May 9;9(5):545. doi: 10.3390/jof9050545 (PMC10219533; doi:10.3390/jof9050545)
Supplement: Supplementary file 1 [file jof-09-00545-s001.zip › Table S4.pdf]

Table S4. Mycoviruses associated with 244 *Rhizoctonia* isolates recovered from sugar beet roots with the symptoms of root and crown rot in China from 2009 to 2016 and the median effective concentration (EC_{50}) of flutolanil, thifluzamide, and pencycuron to these *Rhizoctonia* isolates.

**Isolate code**	**Mycovirus associated *Rhizoctonia*** [z]	**Number of mycovirus**	**EC_{50} (μg·mL^{-1})**		
			**Flutolanil**	**Thifluzamide**	**Pencycuron**
R1	RsMV50, RsNV10	2	0.1210	0.4053	3.0043
R2	RsNV16	1	0.0770	0.0768	4.7210
R3	RsMV92, RsMV101, RsNV16	3	0.1640	0.1538	3.1715
R4	RsPV2B, RsNV16	2	0.0950	0.0272	3.2941
R5	RsNV16	1	0.1300	0.1347	0.0042
R6	RsKV, RsMV61, RsMV70, RsMV71, RsNV16	5	0.3710	0.3162	0.0032
R7	RsMV89, RsNV16	2	0.0960	0.1403	0.0129
R8	RsMV84, RsNV16, RsOLV8, RsPV2B	4	0.3750	0.3535	0.0033
R9	RsMV45, RsMV84, RsNV16	3	0.2840	0.1569	0.0065
R10	RsMV67, RsMV84, RsMV98, RsNV16	4	0.4960	0.0886	0.0109
R11	RsNV16	1	0.1600	0.0704	0.0043
R12	RsMV61, RsNV16	2	0.1580	0.1072	0.0051
R13	RsBLV3, RsNV16	2	0.2350	0.0886	0.0032
R15	RsBLV4, RsBLV6, RsBLV5, RsMV72, RsNV16, RsPV2B	6	0.6170	0.5630	0.0117
R16	RsMV45, RsMV70, RsMV102, RsMV103, RsNV16	5	0.1460	0.1208	0.0035
R17	RsNV16	1	0.0710	0.1428	0.0065
R18	RsNV16, RsOLV7, RsPV2B	3	0.0470	0.1014	0.0150
R19	RsMV57, RsNV16	2	0.2210	0.0669	0.0108
R20	RsNV16	1	0.0220	0.1008	0.0100
R21	RsMV99, RsNV16	2	0.2220	0.0962	0.0057
R23	RsNV16	1	0.1390	0.0640	0.0132
R24	RsNV16	1	0.1310	0.1385	0.0224

Table S4. (Continued from preceding page).

Isolate code	Mycovirus associated *Rhizoctonia* [z]	Number of mycovirus	EC_{50} ($\mu g \cdot mL^{-1}$)		
			Flutolanil	Thifluzamide	Pencycuron
R25	RsMV54, RsNV16	2	0.0700	0.0914	0.0137
R26	RsMV83, RsMV93, RsNV16	3	0.1280	0.1259	0.0157
R28	RsMV66, RsMV90, RsNV16	3	0.2520	0.0446	0.0166
R29	RsNV16, RsPV2B	2	0.3810	0.1414	0.0241
R30	RsKV, RsMV45, RsMV72, RsNV16	4	0.1880	0.0211	0.0097
R32	RsMV45, RsMV58, RsMV87, RsNV16	4	0.2310	0.0231	0.0183
R33	RsMV45, RsMV87, RsNV16	3	0.3750	0.0502	0.0157
R35	RsMV87, RsMV104, RsNV16	3	0.3680	0.0695	0.0167
R36	RsNV16	1	0.0140	0.1150	0.0051
R37	RsMV73, RsMV75, RsMV80, RsNV16	4	0.0830	0.0741	0.0105
R38	RsMV82, RsMV99, RsNV16	3	0.3620	0.0615	0.0052
R39	RsNV16, RsPV17	2	0.0770	0.1069	0.0186
R40	RsMV90, RsNV16	2	0.0570	0.0860	5.4887
R41	RsNV16	1	0.2280	0.1125	0.0118
R42	RsMV57, RsNV16, RsPV2B	3	0.2360	0.3061	0.0100
RR2	RsMV65, RsMV81, RsNV16	3	0.3650	0.6767	27.8497
RR5	RsNV16	1	0.1220	0.0636	5.3201
RR6	RsNV16, RsOLV7	2	0.0520	0.1402	0.0112
RR7	RsMV62, RsMV81, RsMV86, RsMV87, RsMV94, RsMV100, RsNV16	7	0.1230	0.1129	0.0160
RR8	RsMV56, RsNV2, RsNV16	3	0.0940	0.1026	0.0049
RR11	RsNV16	1	0.4260	0.1844	0.0063
RR13	RsNV16	1	0.3760	0.0456	0.0056

Table S4. (Continued from preceding page).

Isolate code	Mycovirus associated *Rhizoctonia* [z]	Number of mycovirus	EC50 (μg·mL⁻¹)		
			Flutolanil	Thifluzamide	Pencycuron
RR16	RsBLV3, RsMV90, RsNV16	3	0.3470	0.1276	0.0059
RR17	RsMV68, RsMV99, RsNV16, RsPV15	4	0.2790	0.1600	0.0068
RR18	RsMV56, RsMV96, RsNV16	3	0.1490	0.1230	0.0093
RR20	RsMV62, RsMV81, RsMV87, RsMV94, RsNV16, RsPV15, RsPV20	7	0.1420	0.0369	2.8348
RR22	RsMV81, RsNV16	2	0.1480	0.0503	3.2472
RR23	RsMV81, RsNV16	2	0.0980	0.0454	3.0694
RR25	RsMV87, RsMV99, RsNV16, RsPV15	4	0.2210	0.1390	0.0074
RR26	RsNV16, RsPV2B	2	0.1640	0.1603	0.0140
RR27	RsNV16	1	0.2220	0.0776	4.5461
R1(12)	RsMV49, RsMV60, RsMV66, RsMV88, RsNV16	5	0.2760	0.0962	0.0081
R2(12)	RsMV47, RsMV48, RsMV59, RsNV16, RsPV2B	5	0.2330	0.0979	0.0109
R3(12)	RsMV87, RsNV16	2	0.1390	0.1113	0.0067
R4(12)	RsMV42, RsMV54, RsMV95, RsNV16	4	0.1330	0.1405	0.0193
R5(12)	RsMV42, RsMV74, RsNV16, RsNLV1	4	0.2140	0.1525	0.0073
R6(12)	RsNV15, RsNV16, RsPV2B, RsPV17, RsPV20	5	0.1230	0.1174	0.0126
RHB-1	RsMV42, RsMV74, RsMV81, RsMV95, RsNV16	5	0.1095	0.0266	5.9142
RHB-2	RsMV42, RsMV74, RsMV95, RsNV16	4	0.1271	0.0452	6.0018
RHB-3	RsMV81, RsNV16	2	0.1253	0.0347	5.8003
RHB-4	RsMV42, RsMV74, RsMV81, RsMV95, RsNV16	5	0.1200	0.0315	5.5436
RHL1	RsMV87, RsNSV7, RsNV13, RsNV16	4	0.0752	0.0421	0.0276
RHL2	RsNSV7, RsNV13, RsNV16	3	0.0687	0.0482	0.0266
RHL3	RsHV8, RsMV53, RsMV87, RsNV16	4	0.0916	0.0588	0.0365

Table S4. (Continued from preceding page).

Isolate code	Mycovirus associated *Rhizoctonia* [z]	Number of mycovirus	EC$_{50}$ (μg·mL^{-1})		
			Flutolanil	Thifluzamide	Pencycuron
RHL4	RsNSV7, RsNV13, RsNV16	3	0.0632	0.0404	0.0315
RHL5	RsMV80, RsMV83, RsNSV7, RsNV13, RsNV16, RsPV20	6	0.0658	0.0502	0.0315
RHL6	RsNV16, RsPV17	2	0.1409	0.0687	0.0244
RNM-1	RsMV42, RsMV74, RsMV81, RsMV87, RsMV95, RsNV16	6	0.1928	0.0689	0.0209
RNM-2	RsMV42, RsMV74, RsMV81, RsMV95, RsNV16	5	0.2030	0.0671	0.0294
RNM-3	RsNV16	1	0.2532	0.0334	0.0287
RNM-4	RsNV16	1	0.2480	0.0332	0.0219
RNM-5	RsMV82, RsMV99, RsMV105, RsNV16, RsPV18, RsPV19	6	0.2047	0.0244	0.0094
RNM-7	RsNV16	1	0.3185	0.0318	0.0174
RNM-8	RsNV16, RsPV15	2	0.3417	0.0583	0.0187
RNM-9	RsMV87, RsNV16, RsPV2B, RsPV16, RsPV19	5	0.4212	0.0536	0.0175
RSX-1	RsBLV4, RsBLV5, RsMV62, RsMV74, RsMV80, RsMV87, RsNV16, RsPV2B	8	0.1448	0.0725	0.0156
RSX-2	RsBLV4, RsMV62, RsMV80, RsMV87, RsNV16, RsPV2B	6	0.0819	0.0492	0.0092
RSX-3	RsNV16	1	0.1102	0.0419	0.0158
RSX-4	RsBLV4, RsBLV6, RsBLV5, RsMV80, RsNV16	5	0.1307	0.0488	0.0161
RSX-5	RsBLV4, RsBLV5, RsMV73, RsMV75, RsMV80, RsNV16, RsPV2B	7	0.0969	0.0431	0.0140
RSX-6	RsBLV4, RsBLV6, RsBLV5, RsMV80, RsNV16, RsPV2B	6	0.1727	0.0465	0.0204
RSX-7	RsNV16	1	0.1200	0.0522	0.0133
RX-1	RsNV16	1	0.1618	0.0673	0.0175
RX-2	RsMV42, RsMV74, RsMV95, RsNV16	4	0.2051	0.1116	0.0342
RX-3	RsNV16	1	0.1156	0.0333	0.0210
RX-4	RsMV55, RsNV16	2	0.1576	0.0402	0.0257

Table S4. (Continued from preceding page).

Isolate code	Mycovirus associated *Rhizoctonia* [z]	Number of mycovirus	EC50 (μg·mL^{-1})		
			**Flutolanil**	**Thifluzamide**	**Pencycuron**
RX-5	RsBLV2, RsBLV4, RsBLV5, RsMV55, RsMV73, RsNV10, RsNV16, RsPV2B	8	0.1458	0.0524	0.0240
RX6	RsHV5, RsHV7, RsNV2, RsNV16, RsPV2B	5	0.1795	0.1201	0.0153
RX7	RsHV5, RsHV7, RsMV42, RsMV74, RsMV95, RsNV16	6	0.1960	0.0974	0.0166
RX8	RsHV5, RsNV2, RsNV16	3	0.1918	0.0935	0.0214
RX9	RsBLV2, RsBLV3, RsNV16	3	0.1035	0.0616	0.0147
RX10	RsBLV2, RsBLV3, RsNV16	3	0.1189	0.0780	0.0145
RX11	RsBLV2, RsBLV3, RsBLV4, RsBLV5, RsBLV6, RsMV83, RsNV16	7	0.1600	0.0900	0.0140
RX12	RsBLV2, RsBLV3, RsBLV4, RsBLV5, RsBLV6, RsMV83, RsNV16	7	0.1163	0.0729	0.0186
RX13	RsNV16	1	0.1275	0.0823	0.0202
RX14	RsBLV3, RsMV42, RsMV83, RsNV16	4	0.1347	0.0835	0.0210
RX15	RsMV64, RsNV16	2	0.1353	0.0756	0.0235
RX16	RsBLV2, RsBLV3, RsMV83, RsNV16	4	0.1230	0.0777	0.0227
RX17	RsBLV2, RsBLV3, RsNV16	3	0.1220	0.0823	0.0215
RN5	RsNV16	1	0.1155	0.1001	0.0359
RN6	RsNV16	1	0.2997	0.0699	0.0601
RN10	RsMV63, RsMV99, RsNV16	3	0.4189	0.1126	0.0546
RXJ3	RsNV16	1	0.1222	0.1015	0.0541
RHL9	RsMV75, RsNV16	2	0.0938	0.0498	0.0466
RHL10	RsMV75, RsNV16	2	0.0803	0.0734	0.0493
RHL11	RsMV75, RsNV16	2	0.0837	0.0681	0.0450
RHL17	RsNV16	1	0.1531	0.0791	0.0417
RN11	RsNV16	1	0.1288	0.0476	0.0238

Table S4. (Continued from preceding page).

Isolate code	Mycovirus associated *Rhizoctonia* [z]	Number of mycovirus	EC$_{50}$ (μg·mL^{-1})		
			Flutolanil	Thifluzamide	Pencycuron
RN15	RsNV16	1	0.5647	0.1126	0.0408
RN17	RsMV51, RsNV16	2	0.2889	0.0928	10.9504
RN19	RsMV51, RsNV16	2	0.3512	0.0850	14.4323
RN20	RsNV16, RsPV2B	2	0.2632	0.0526	0.0418
RN21	RsMV51, RsNV16, RsPV15	3	0.2271	0.1205	0.0328
RN22	RsNV16, RsOLV8, RsPV2B	3	0.2393	0.1087	0.0318
RN23	RsNV16	1	0.2976	0.1352	0.0372
RN25	RsNV16	1	0.2154	0.1215	0.0364
RN28	RsNV16	1	0.7893	0.1871	0.0425
RN29	RsNV16	1	0.6872	0.1162	0.0368
RN31	RsNV16	1	0.6059	0.1210	0.0437
RN39	RsMV56, RsMV77, RsMV86, RsNV16, RsPV2B	5	0.5200	0.0818	0.0393
RN43	RsNV16	1	0.8706	0.1818	0.0474
RN44	RsNV16	1	1.1748	0.1436	0.0721
RN49	RsBLV3, RsMV45, RsNV16	3	0.8926	0.1832	0.0521
RN50	RsNV16	1	1.0102	0.1828	0.0512
RN53	RsNV16, RsPV2B	2	1.0231	0.1924	0.0706
RN56	RsMV45, RsNV16	2	1.0589	0.1695	0.0628
RN57	RsNV16	1	0.7034	0.1598	0.0606
RN60	RsMV45, RsNV16	2	1.1904	0.1306	0.0675
RN61	RsMV45, RsNV2, RsNV16, RsPV2B	4	0.6312	0.1206	0.0515
RN62	RsNV16, RsPV2B	2	0.7140	0.1071	0.0434

Table S4. (Continued from preceding page).

Isolate code	Mycovirus associated *Rhizoctonia* [z]	Number of mycovirus	EC $_{50}$ (μg·mL $^{-1}$)		
			Flutolanil	Thifluzamide	Pencycuron
RN63	RsNV16	1	0.6276	0.1083	0.0383
RN65	RsNV16	1	0.5596	0.1216	0.0417
RN66	RsNV16	1	0.7664	0.1537	0.0483
RN67	RsNV16	1	0.6381	0.1311	0.0579
RN69	RsNV16	1	0.5384	0.1217	0.0488
RN72	RsNV16	1	0.5895	0.2443	0.0420
RN74	RsNV16, RsPV2B	2	0.6700	0.1218	0.0411
RN76	RsMV56, RsMV77, RsMV86, RsNV16	4	0.5794	0.0801	0.0313
RN78	RsNV16	1	0.1844	0.0705	0.0238
RN79	RsNV16	1	0.2254	0.1048	0.0280
RN80	RsNV16	1	0.2320	0.1227	0.0370
RN81	RsNV16	1	0.2322	0.1168	0.0316
RN82	RsNV16	1	0.2506	0.1337	0.0318
RN83	RsMV76, RsNV16	2	0.1854	0.0845	0.0338
RN84	RsMV62, RsMV65, RsMV83, RsMV94, RsNV16	5	0.1895	0.1156	0.0394
RN85	RsNV16	1	0.2346	0.1052	0.0372
RN86	RsMV76, RsNV16	2	0.2336	0.1043	0.0348
RN87	RsNV16	1	0.2684	0.1655	0.0356
RN88	RsMV76, RsNV16	2	0.2043	0.1133	0.0320
RN90	RsMV76, RsNV16	2	0.2488	0.1401	0.0373
RN91	RsMV99, RsNV16	2	0.5895	0.0801	0.0403
RN92	RsNV16	1	0.5963	0.1220	0.0459

Table S4. (Continued from preceding page).

Isolate code	Mycovirus associated *Rhizoctonia* [z]	Number of mycovirus	EC$_{50}$ (μg·mL^{-1})		
			**Flutolanil**	**Thifluzamide**	**Pencycuron**
RN94	RsNV16	1	0.7662	0.1641	0.0635
RN95	RsNV16	1	0.6885	0.1245	0.0586
RN97	RsMV44, RsNV16	2	0.6830	0.0796	0.0400
RN98	RsMV99, RsNV16	2	0.6971	0.0875	0.0402
RN99	RsNV16	1	0.6236	0.1080	0.0396
RN100	RsNV16, RsPV2B	2	0.6352	0.0881	0.0431
RN102	RsNV16	1	0.4588	0.0807	0.0426
RN104	RsMV41, RsMV76, RsNV16	3	0.6418	0.0883	0.0418
RN105	RsNV16	1	0.1997	0.0831	0.0328
RX18	RsMV83, RsNV16	2	0.1774	0.1081	0.0516
RX19	RsMV75, RsMV83, RsNV16	3	0.1575	0.0962	0.0568
RX20	RsMV75, RsMV77, RsMV83, RsNV16	4	0.1756	0.0961	0.0518
RX21	RsMV75, RsMV83, RsNV16, RsPV2B	4	0.1637	0.0935	0.0410
RX22	RsNV16	1	0.1556	0.0989	0.0403
RX23	RsHV8, RsMV83, RsNV16	3	0.1140	0.1092	0.0304
RX24	RsMV42, RsMV95, RsNV16	3	0.1945	0.1367	0.0401
RX25	RsMV42, RsMV87, RsMV94, RsMV95, RsNV16	5	0.2137	0.1234	0.0548
RX26	RsMV77, RsMV95, RsNV16, RsPV2B	4	0.2058	0.0952	0.0402
RX28	RsNV16	1	0.3726	0.1332	0.0228
RX29	RsNV16	1	0.3228	0.0741	0.0434
RX31	RsNV16, RsPV2B	2	0.4138	0.0625	0.0387
RX39	RsMV41, RsMV76, RsMV91, RsNV16, RsPV2B	5	0.1795	0.0985	0.0411

Table S4. (Continued from preceding page).

Isolate code	Mycovirus associated *Rhizoctonia* [z]	Number of mycovirus	EC50 (μg·mL⁻¹)		
			Flutolanil	Thifluzamide	Pencycuron
RX44	RsMV41, RsMV76, RsMV91, RsNV16	4	0.1565	0.0895	0.0317
RN106	RsHV8, RsNV16	2	0.2180	0.1198	0.0547
RN107	RsMV44, RsNV16, RsPV17	3	0.2069	0.1350	0.0528
RN108	RsHV8, RsMV46, RsNV16, RsPV17	4	0.2307	0.0993	0.0465
RN109	RsHV8, RsNV16	2	0.1930	0.1377	0.0446
RN110	RsNV16, RsPV17	2	0.2624	0.1206	0.0530
RN111	RsBLV3, RsHV8, RsNV16	3	0.2348	0.1156	0.0489
RN112	RsHV8, RsNV16	2	0.2356	0.1632	0.0555
RN113	RsHV8, RsNV16	2	0.2651	0.1603	0.0526
RN114	RsMV72, RsNV16, RsPV16	3	0.7715	0.1753	0.0485
RN115	RsMV62, RsMV81, RsMV86, RsMV94, RsNV16	5	0.3640	0.0619	0.0538
RN116	RsNV16	1	0.6074	0.0839	0.0658
RN117	RsNV16	1	0.6762	0.1870	0.0493
RN118	RsNV16	1	0.5867	0.0931	0.0561
RN119	RsBLV3, RsNV16	2	0.6703	0.0965	0.0547
RN120	RsNV16	1	0.4560	0.0974	0.0378
RN121	RsKV, RsMV72, RsNV16, RsOLV8, RsPV2B	5	0.3646	0.0885	0.0513
RN122	RsBLV3, RsNV16, RsOLV8	3	0.2322	0.0703	0.0380
RN123	RsKV, RsNV16, RsOLV8	3	0.4603	0.0792	0.0436
RN124	RsNV16, RsOLV8	2	0.2670	0.0577	0.0409
RN125	RsNV16	1	0.5645	0.1015	0.0514
RN126	RsNV16, RsOLV8	2	0.5594	0.0851	0.0485

Table S4. (Continued from preceding page).

Isolate code	Mycovirus associated *Rhizoctonia* [z]	Number of mycovirus	EC_{50} (μg·mL^{-1})		
			**Flutolanil**	**Thifluzamide**	**Pencycuron**
RN127	RsBLV3, RsNV16, RsOLV8, RsPV17	4	0.4400	0.1075	0.0453
RN128	RsNV16, RsOLV8	2	0.3703	0.1013	0.0386
RN129	RsNV16, RsPV16, RsPV20, RsPV21	4	0.3424	0.0633	0.0374
RN130	RsMV69, RsNV16, RsPV21	3	0.5125	0.0873	0.0388
RN131	RsMV69, RsNV16, RsPV16, RsPV20, RsPV21	5	0.2641	0.0650	0.0358
RN132	RsMV69, RsNV16, RsPV16, RsPV21	4	0.3943	0.0976	0.0405
RN133	RsHV8, RsMV74, RsNV16	3	0.2112	0.1067	0.0549
RN134	RsMV42, RsMV74	2	0.1596	0.0873	0.0478
RN135	RsMV41, RsMV76, RsMV91, RsNV1, RsNV3, RsNV8, RsNV16	7	0.2360	0.1323	0.0514
RN136	RsBLV4, RsBLV5, RsHV8, RsMV41, RsMV76, RsMV91, RsNV1, RsNV4, RsNV8, RsNV9, RsNV12, RsNV16, RsNV18	13	0.1948	0.1307	0.0440
RN137	RsBLV3, RsBLV4, RsBLV5, RsHV8, RsMV41, RsMV76, RsMV91, RsNV1, RsNV3, RsNV4, RsNV6, RsNV7, RsNV8, RsNV9, RsNV12, RsNV14, RsNV16, RsNV18	18	0.2260	0.1077	0.0502
RN138	RsBLV4, RsBLV5, RsHV8, RsMV41, RsMV76, RsMV91, RsNLV2, RsNV1, RsNV3, RsNV4, RsNV8, RsNV11, RsNV12, RsNV16, RsNV18, RsPV15, RsPV17	17	0.2106	0.1551	0.0508
RN139	RsNLV2, RsNV1, RsNV3, RsNV8, RsNV11, RsNV13, RsNV16	7	0.2256	0.1299	0.0581
RN140	RsBLV3, RsNLV2, RsNV1, RsNV3, RsNV4, RsNV8, RsNV11, RsNV16, RsPV17	9	0.2432	0.1628	0.0472
RN141	RsBLV4, RsBLV5, RsHV8, RsMV41, RsMV76, RsMV91, RsNV1, RsNV4, RsNV5, RsNV6, RsNV7, RsNV8, RsNV9, RsNV12, RsNV14, RsNV16, RsNV17, RsNV18, RsNV19, RsPV17	20	0.1103	0.0599	0.0407
RN142	RsBLV4, RsBLV5, RsHV8, RsNV16, RsPV17	5	0.2287	0.0975	0.0561
RN143	RsMV42, RsMV74, RsNV16, RsPV20	4	0.1283	0.0996	0.0365

Table S4. (Continued from preceding page).

Isolate code	Mycovirus associated *Rhizoctonia* [z]	Number of mycovirus	EC50 (μg·mL⁻¹)		
			Flutolanil	Thifluzamide	Pencycuron
RN144	RsMV42, RsMV74, RsNV16, RsPV20	4	0.1349	0.1069	0.0346
RN145	RsNV16, RsPV17	2	0.2227	0.1332	0.0394
RN146	RsMV43, RsMV56, RsMV78, RsMV86, RsNV16, RsPV2B, RsPV16, RsPV18, RsPV19	9	0.4852	0.1404	0.0508
RN147	RsMV43, RsMV56, RsMV78, RsMV86, RsNV16, RsPV2B, RsPV18, RsPV19	8	0.7049	0.0934	0.0510
RN148	RsBLV3, RsMV43, RsMV56, RsMV77, RsMV78, RsNV16, RsPV16, RsPV18, RsPV19	9	0.1972	0.1158	0.0420
RN149	RsHV8, RsMV43, RsMV56, RsMV77, RsMV78, RsMV86, RsNV16, RsPV16, RsPV18, RsPV19	10	0.5552	0.0929	0.0540
RN150	RsBLV3, RsMV43, RsMV56, RsMV78, RsMV86, RsNV16, RsPV16, RsPV18, RsPV19	9	0.4130	0.1659	0.0539
RN151	RsNV16, RsPV19	2	0.6774	0.1470	0.0621
RN152	RsMV56, RsNV16, RsPV18, RsPV19	4	0.3974	0.0621	0.0326
RN153	RsMV56, RsNV16, RsPV16, RsPV18, RsPV19	5	0.8131	0.1108	0.0539
RN154	RsNV16, RsPV2B, RsPV16, RsPV19	4	0.4132	0.0703	0.0478
RN155	RsNV16, RsPV16, RsPV19	3	0.3376	0.0530	0.0304
RN156	RsMV43, RsMV56, RsMV77, RsNV16, RsPV2B, RsPV19	6	0.5455	0.1327	0.0283
RN157	RsNV16, RsPV2B, RsPV16, RsPV19	4	0.5651	0.1157	0.0507
RN158	RsMV77, RsNV16, RsPV16, RsPV19	4	0.4940	0.1770	0.0525
RN159	RsMV77, RsNV16, RsPV16, RsPV18, RsPV19	5	0.5351	0.2165	0.0605
RN160	RsBLV3, RsNV16, RsPV16, RsPV18, RsPV19	5	0.4777	0.0687	0.0374
RN161	RsNV16, RsPV16, RsPV19	3	0.6307	0.1391	0.0504
RN162	RsNV16, RsPV16, RsPV19	3	0.5906	0.1666	0.0668
RN163	RsBLV2, RsNV16, RsPV19	3	0.8279	0.1392	0.0575

Table S4. (Continued from preceding page).

Isolate code	Mycovirus associated *Rhizoctonia* [z]	Number of mycovirus	EC_{50} (μg·mL^{-1})		
			**Flutolanil**	**Thifluzamide**	**Pencycuron**
RN164	RsMV41, RsMV69, RsMV76, RsMV91, RsNV16, RsPV2B	6	0.3943	0.0744	0.0361
RN165	RsBLV2, RsMV41, RsMV69, RsMV76, RsMV91, RsMV99, RsNV16, RsPV19	8	0.5619	0.1180	0.0767
RN166	RsBLV2, RsMV41, RsMV76, RsNV16, RsPV19	5	0.5810	0.0964	0.0479
RN167	RsMV42, RsMV74, RsMV95, RsNV16, RsPV16, RsPV17	6	0.1048	0.0474	6.7826
RN168	RsMV42, RsMV74, RsMV81, RsMV95, RsNV16	5	0.0853	0.0752	7.8470
RN169	RsMV42, RsMV74, RsMV81, RsMV95, RsNV16, RsPV16	6	0.1568	0.0714	3.6373
RX46	RsNV2, RsNV16, RsPV2B	3	0.5370	0.1076	0.0549
RX47	RsMV41, RsMV46, RsMV52, RsMV76, RsMV85, RsMV91, RsMV99, RsNV16	8	0.5533	0.0951	0.0442
RX48	RsHV6, RsHV7, RsMV46, RsMV52, RsMV76, RsMV85, RsMV91, RsNV2, RsNV16, RsPV2B	10	0.6919	0.1411	0.0643

Note: [z] Letters followed by Arabic numerals represent the abbreviation of the mycovirus. RsMV, Rhizoctonia solani mitovirus; RsNV, Rhizoctonia solani narnavirus; RsPV, Rhizoctonia solani partitivirus; RsKV, Rhizoctonia solani Khurdun virus; RsOLV, Rhizoctonia solani ourmia-like virus; RsBLV, Rhizoctonia solani beny-like virus; RsHV, Rhizoctonia solani hypovirus; RsNSV, Rhizoctonia solani negative-stranded virus; RsNLV, Rhizoctonia solani narna-like virus.
